# ROR1 regulates chemoresistance in Breast Cancer via modulation of drug efflux pump ABCB1

**DOI:** 10.1038/s41598-020-58864-0

**Published:** 2020-02-04

**Authors:** Norman Fultang, Abhinav Illendula, Jianhuang Lin, Manoj K. Pandey, Zachary Klase, Bela Peethambaran

**Affiliations:** 1 Department of Biological Sciences, University of the Sciences in Philadelphia, Philadelphia, Pennsylvania 19104 United States of America; 2 The Wistar Institute, Philadelphia, Pennsylvania 19104 United States of America; 3 Department of Biomedical Sciences, Cooper Medical School of Rowan University, Camden, New Jersey 08103 USA

**Keywords:** Breast cancer, Cell signalling

## Abstract

Chemoresistance is one of the leading causes of mortality in breast cancer (BC). Understanding the molecules regulating chemoresistance is critical in order to combat chemoresistant BC. Drug efflux pump ABCB1 is overexpressed in chemoresistant neoplasms where it effluxes various chemotherapeutic agents from cells. Because it is expressed in normal and cancerous cells alike, attempts at targeting ABCB1 directly have failed due to low specificity and disruption of normal tissue. A proposed method to inhibit ABCB1 is to target its cancer-specific, upstream regulators, mitigating damage to normal tissue. Few such cancer-specific upstream regulators have been described. Here we characterize ROR1 as an upstream regulator of ABCB1. ROR1 is highly expressed during development but not expressed in normal adult tissue. It is however highly expressed in several cancers. ROR1 is overexpressed in chemoresistant BC where it correlates with poor therapy response and tumor recurrence. Our data suggests, ROR1 inhibition sensitizes BC cells to chemo drugs. We also show ROR1 regulates ABCB1 stability and transcription via MAPK/ERK and p53. Validating our overall findings, inhibition of ROR1 directly correlated with decreased efflux of chemo-drugs from cells. Overall, our results highlight ROR1’s potential as a therapeutic target for multidrug resistant malignancies.

## Introduction

Breast cancer is the leading cause of cancer-related deaths in women worldwide^1^. Poor survival rates and therapy failure in breast cancer are attributable to several factors most notably chemoresistance. Chemoresistance is a phenomenon wherein tumors exhibit resistance to the anticancer effects of chemotherapeutic agents^2^. These chemoresistant tumors often require unbearably high therapeutic doses with disastrous side effects for patients^3^.

Understanding the molecular mechanisms underlying chemoresistance is critical in order to combat chemoresistant malignancies.

The ABC family of ATP-dependent drug effluxers, most notably ABCB1/MDR-1, have been shown to play a major role in chemoresistance^2,4,5^. ABCB1 can transport a variety of substrates out of cells via ATP-hydrolysis^2,5,6^. In cancer, ABCB1 actively effluxes a broad range of chemotherapeutic agents from cells leading to multidrug resistance^6^. Attempts at overcoming multidrug resistance using inhibitors for ABCB1 and other drug effluxers have had disappointing outcomes clinically due to low specificity and disruption of normal tissue^7^.

A proposed method to selectively target ABCB1 and chemoresistance in breast cancer has been to target cancer-specific upstream regulators of ABCB1, minimizing any detrimental effects to normal tissue. To this aim, the identification and characterization of upstream regulators of ABCB1, highly expressed in cancers but only minimally expressed in normal cells, is necessary.

Here we identify ROR1 as a key upstream regulator of ABCB1 and chemoresistance in breast cancer. ROR1 is an oncofetal receptor highly expressed in several human malignancies but not expressed in normal adult tissue^8–10^. Previous work has shown ROR1 to be enriched in chemoresistant stem cells and be a prognostic marker for relapse and poor therapeutic outcomes^8,11–15^. Analysis of breast cancer patient gene expression data from NCBI’s Gene Expression Omnibus (GEO) suggests ROR1 is enriched in patients with poor response to chemotherapy. Our results suggest ROR1 knockdown, or inhibition with a novel small molecule inhibitor, potentiates the effect of Pt-based and anthracycline chemo drugs. We also found ROR1 to promote ABCB1 stability by repressing its proteasomal degradation in a MAPK/ERK-dependent manner. On a transcriptional level, we observed a decrease in ABCB1 mRNA after ROR1 inhibition and knockdown. Chromatin immunoprecipitation of regulators bound to the ABCB1 promoter suggested increased p53 and SMARCB1 at the ABCB1 promoter (two ABCB1 transcriptional repressors usually inhibited by ROR1) after ROR1 knockdown and inhibition. Validating our overall findings, inhibition of ROR1 directly correlated with decreased efflux of chemo-drugs from cells. Overall, our results describe the mechanism by which ROR1 regulates efflux drug pump ABCB1. They also highlight ROR1’s potential as a therapeutic target for multidrug resistant malignancies.

## Results

### ROR1 is enriched in chemoresistant tumors and cell lines

**Figure 1:**
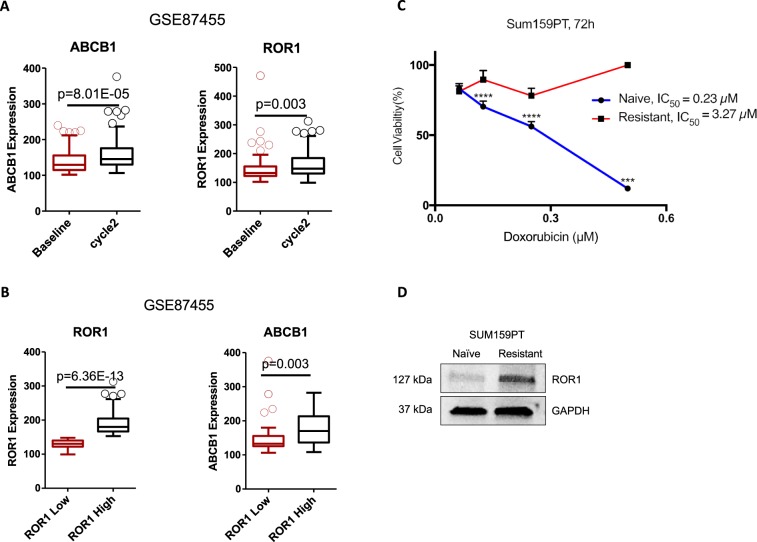
ROR1 is overexpressed in chemoresistant breast cancer and enriched post chemotherapy. (**A**) ROR1 and ABCB1 expression levels in matched breast cancer patient samples pre- (baseline) or post- (cycle 2) chemotherapy. (**B**) ROR1 and ABCB1 expression levels in ROR1-high (higher than median) and ROR1-low (lower than median) groups investigating correlation between ROR1 and ABCB1. N = 57, Accession number = GSE87455. (**C**) MTT assessing cell viability of SUM159PT/R (resistant) and SUM159PT (naïve) after treatment with Dox for 72 h. (**D**) Immunoblot assessing ROR1 expression in SUM159PT/R (resistant) and SUM159PT (naïve) cells. Full length blots are presented in the Supplementary Fig. S7. Statistical analyses performed via student’s t test. ****p < 0.0001, ***p < 0.001.

Given the fact that ROR1 has been shown to be a prognostic marker for relapse and poor therapeutic outcomes, we explored whether ROR1 is involved in chemoresistance. To this aim, data mining was conducted based on GSE87455 dataset^16^. Gene expression of ROR1 was compared between pre- and post-chemotherapy in patients with HER2-negative breast cancer. Along with basal BC, the HER-2 enriched group is the other BC subtype where the development of chemoresistance is most frequently observed^17,18^. ABCB1 was used as a marker for chemoresistance (Fig. 1A). ABCB1 expression was significantly increased in patients who received 2 cycles of chemotherapy “Cycle 2” compared to “Baseline” expression pre-chemo, suggesting the development of chemoresistance after chemotherapy. Interestingly, ROR1 expression was also significantly upregulated in the post-chemotherapy group, indicating that ROR1 may be involved in the development of chemoresistance. To investigate whether the upregulated ROR1 correlated with increased ABCB1 expression, we divided the patients into ROR1 -low and -high groups using the median expression as a cutoff (Fig. 1B). Notably, ABCB1 expression was significantly upregulated in ROR1-high group, indicating that ABCB1 expression correlates with ROR1 expression. This suggests ROR1 may regulate the expression of ABCB1. We validated the data mining results in an *in vitro* system using a multidrug resistant SUM-159PT cell line (SUM-159PT/R). These cells were developed by selection of surviving cells following sequential treatment with Paclitaxel and maintained in media containing either Paclitaxel or Doxorubicin, in an alternating manner. We first validated the chemoresistant phenotype by MTT following Doxorubicin treatment. We observed a 14-fold increase in the IC50 of Doxorubicin in SUM-159PT/R when compared to naive SUM-159PT (3.266 µM and 0.2291 µM, respectively) (Fig. 1C). We then probed ROR1 expression in both resistant and naive cells via immunoblot and observed an increase in ROR1 expression in the resistant cells (Fig. 1D). Altogether, these data suggest ROR1 is enriched in chemoresistant breast cancer tumors and cell lines.

### ROR1 modulation regulates chemoresponse in breast cancer *in vitro*

**Figure 2:**
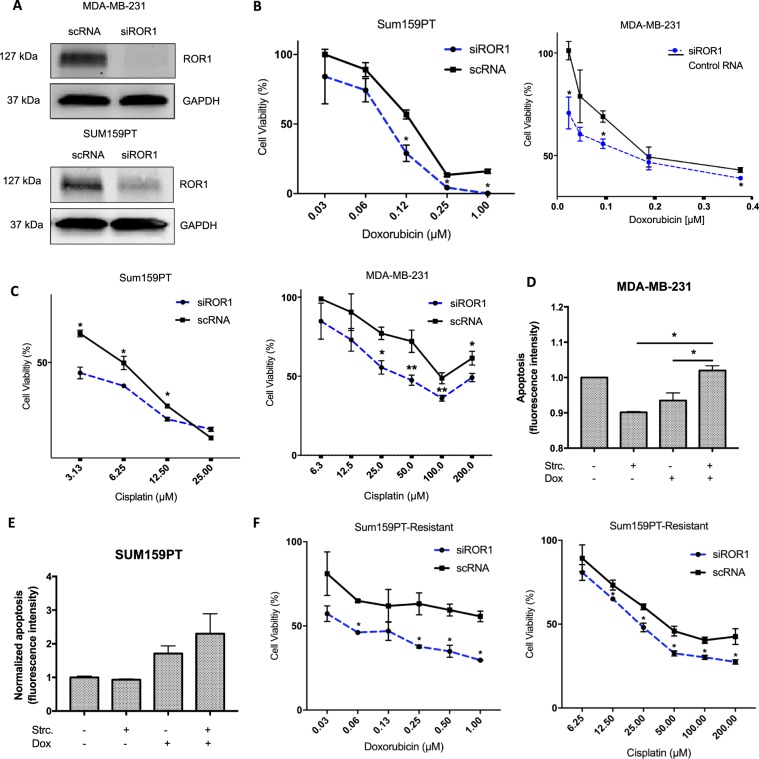
ROR1 inhibition sensitizes BC cells to chemo drugs. (**A**) Immunoblot assessing efficacy of ROR1 knockdown via siRNA in MDA-MB-231 and SUM159PT. Full length blots are presented in the Supplementary Fig. S8. (**B**,**C**) MTT investigating cell viability following Dox/Cis treatment in both cell lines after either siROR1 or Control RNA transfection. (**D**,**E**) Fluorescence-based Annexin-V staining assay to assess apoptosis induction in both cell lines after treatment with Dox/Cis and/or StrC. (**F**) MTT investigating cell viability following Dox/Cis treatment in multidrug-resistant SUM159PT/R after either siROR1 or Control RNA transfection. Statistical analyses via student’s t test. N = 3. *p < 0.05.

To investigate if ROR1 inhibition could potentiate the cytotoxicity induced by chemo drugs *in vitro*, we knocked down ROR1 via siRNA (Fig. 2A) and treated BC lines MDA-MB-231 and SUM-159 PT with Doxorubicin and Cisplatin. We then performed an MTT cell viability assay. For both scRNA and siROR1 groups, cell viability was normalized to a corresponding vehicle treatment control, eliminating any cytotoxicity as a result of transfection methods. We observed an increase in drug-induced cytotoxicity in both cell lines following ROR1 knockdown (Fig. 2B,C). In an ROR1-deficient cell line, MCF-7, we observed a decrease in drug-induced cytotoxicity after transfection with an ROR1-overexpressing plasmid (Supplementary Fig. 1). To further corroborate these findings, we assessed apoptosis induction after treatment with Cis and Dox, with or without an ROR1 inhibitor. We previously described Strictinin (Strc), a naturally-occurring polyphenol as a potent ROR1 inhibitor^10^. We observed an increase in apoptosis in cells treated with the StrC+ drug combination when compared to both treatments individually (Fig. 2D,E). To further validate these findings, we assessed if ROR1 inhibition would reverse the chemoresistant phenotype in the multri-drug resistant SUM-159 PT/R line. We similarly knockdown ROR1 via siRNA and treated the resistant cells with Doxorubicin and Cisplatin. We observed an increase in drug-induced cytotoxicity following ROR1 knockdown indicative of increased chemosensitivity (Fig. 2F, Supplementary Fig. 1a). Altogether, these data suggest that ROR1 modulation regulates chemo drug efficacy in breast cancer cells *in vitro*.

### ROR1 knockdown potentiates DNA damage induced by chemo drugs

**Figure 3:**
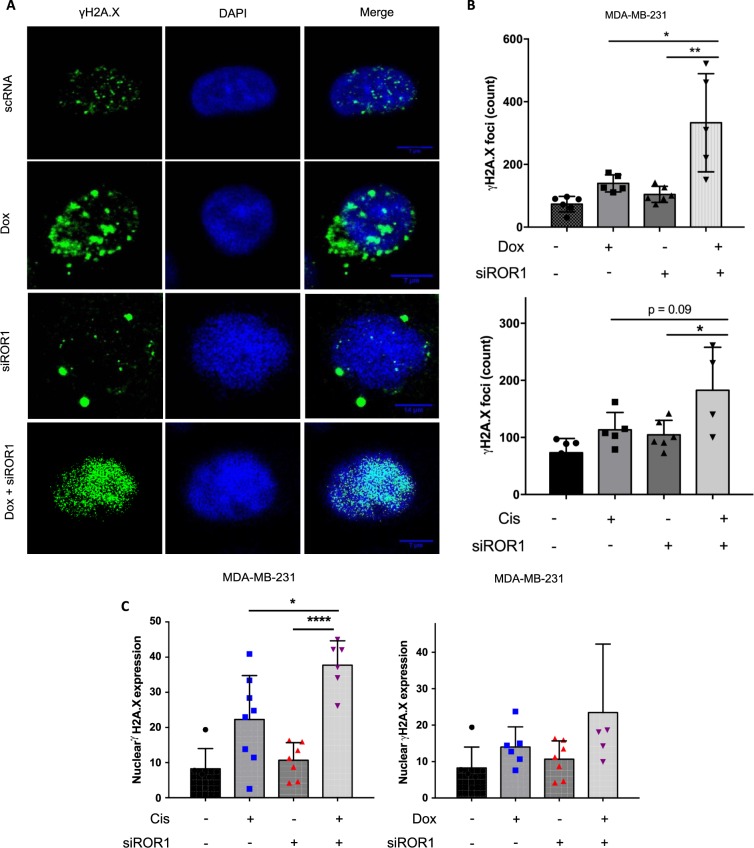
ROR1 inhibition potentiates DNA damage induced by chemo drugs. (**A**) Immunofluorescence labelling γH2A.X in MDA-MB-231 transfected with either siROR1 or control RNA then treated with Dox or vehicle control (DMSO). (**B**) ImageJ quantification of individual γH2A.X foci in nuclei from images obtained in (**A**). (**C**) Overall nuclear γH2A.X expression (mean fluorescence intensity), calculated using ImageJ, of cells imaged in **(A)**. Statistical analyses were performed using Student’s t test. *p < 0.05, **p < 0.01.

A mechanism of action common to both Pt-based and anthracycline chemotherapeutic agents is induction of DNA double stranded breaks leading to cell death^19^. We thus sought to investigate if ROR1 inhibition would promote chemo-induced DNA double strand breaks. We treated cells transfected with either ROR1 siRNA or control RNA, with Doxorubicin or Cisplatin, and monitored γH2a.x, a marker for DNA double strand breaks via immunofluorescence (Fig. 3A, Supplementary Fig. 2). We observed potentiation of DNA double strand breaks induced by both drugs in cells where ROR1 was knocked down. γH2a.x foci counts were higher in the siROR1+ (Dox or Cis) groups compared to the siROR1-only and drug-only treatment groups (Fig. 3B). We similarly observed an increase in γH2a.x expression (mean fluorescence intensity) in Cis/Dox treated cells after ROR1 knockdown compared to the control group (Fig. 3C). Altogether, these data suggest ROR1 inhibition promotes chemo drug-induced DNA damage.

### ROR1 regulates ABCB1 protein and mRNA

**Figure 4:**
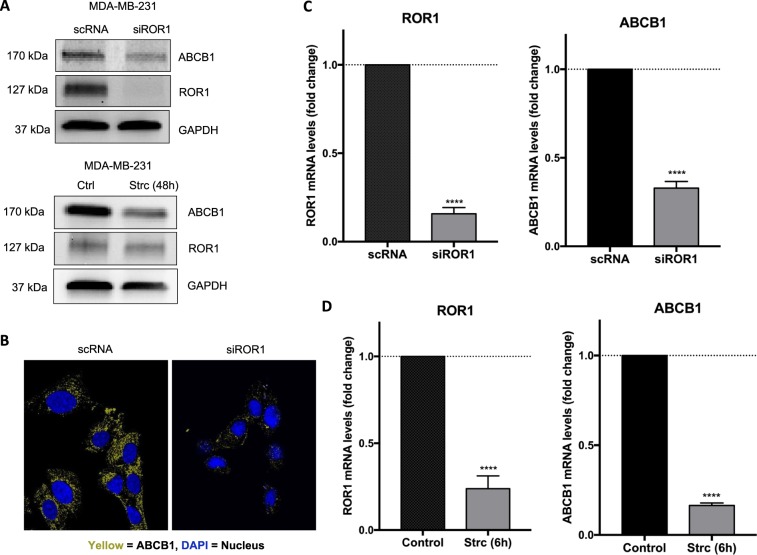
ROR1 regulates ABCB1 protein and mRNA expression. (**A**) Immunoblot assessing ROR1 and ABCB1 protein expression after ROR1 knockdown via siRNA (top panel) or ROR1 inhibition with StrC (bottom panel) in MDA-MB-231. Full length blots are presented in the Supplementary Fig. S9. (**B**) Immunofluorescence labelling ABCB1 in SUM-159PT/R cells transfected with either siROR1 or scRNA (**C**,**D**) qPCR investigating ROR1 and ABCB1 mRNA levels in MDA-MB-231 following ROR1 knockdown via siRNA (**B**) or inhibition with StrC (**C**). Statistical analyses via student’s t test. N = 3. ****p < 0.0001.

To establish a potential mechanism of ROR1 regulation of drug efficacy in breast cancer cells, we investigated the multi-drug efflux pump ABCB1 after ROR1 inhibition. Immunoblots revealed downregulation of ABCB1 protein after either ROR1 knockdown or treatment with Strc (Fig. 4A, Supplementary Fig. 3). Immunofluorescence revealed a decrease in both membrane and cytoplasmic ABCB1 after siROR1 (Fig. 4b). We also observed a significant decrease in ABCB1 mRNA in cells after ROR1 knockdown and Strictinin treatment (Fig. 4C,D). On the other hand, ROR1 overexpression in an ROR1-deficient line, MCF-7, increased ABCB1 (Supplementary Fig. 4).

### ROR1 knockdown reduces ABCB1 protein stability by repressing MAPK/ERK activity

**Figure 5:**
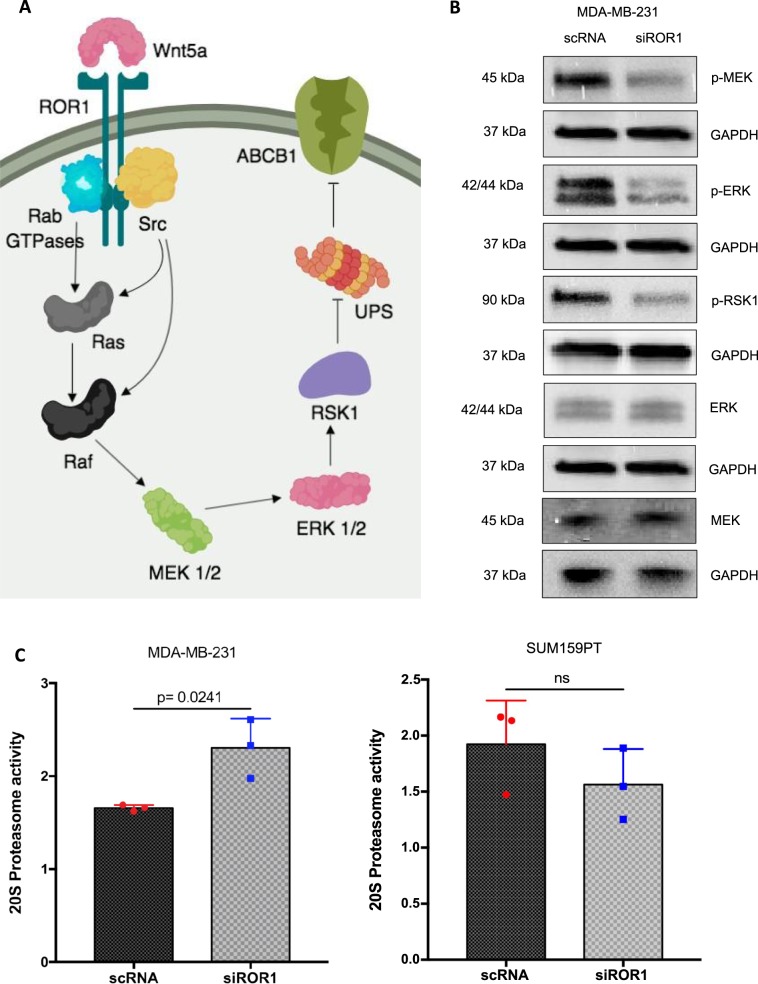
ROR1 promotes ABCB1 stability by repressing UPS degradation via MAPK/ERK. (**A**) The ROR1/MAPK/ERK/ABCB1 signaling pathway. (**B**) Immunoblots investigating expression of proteins in the MAPK/ERK pathway after ROR1 knockdown in MDA-MB-231. Full length blots are presented in the Supplementary Figs. S10 and S11 (**C**) Fluorescence-based assay monitoring activity of the 20 S catalytic subunit of the UPS system which typically degrades ABCB1. Statistical analyses via student’s t test. N = 3. p < 0.05 considered statistically significant.

To investigate the mechanistic link between ROR1 signaling and ABCB1 expression, we monitored pathways regulating ABCB1 degradation and transcription. The MAPK/ERK pathway was previously shown to promote ABCB1 stability by repressing the latter’s degradation by the ubiquitin-proteasome system (UPS)^20,21^. ROR1 signaling was previously shown to activate MAPK/ERK signaling^22^ (Fig. 5A). Using immunoblots, we confirmed a decrease in MEK phosphorylation at Ser217/221, indicative of reduced MEK activation (Fig. 5b). We also observed a decrease in phosphorylation of downstream kinases ERK and p90RSK (RSK1) at residues Thr202/Tyr204 and Ser380 similarly indicative of reduced activation (Fig. 5B). To validate if MAPK/ERK repression correlated with increased UPS activity, we monitored proteasome activity of the 20 S proteasome core subunit after ROR1 inhibition. We observed an increase in proteasome activity after ROR1 knockdown when compared to the control RNA group (Fig. 5C). In SUM159PT, however, changes in 20 S proteasome activity were not significant (Fig. 5C). These data suggest ROR1 inhibition represses MAPK/ERK activity and the latter’s inhibition of the UPS.

### ROR1 inhibition promotes p53 activity and p53/SMARCB1 repression of ABCB1 transcription

**Figure 6:**
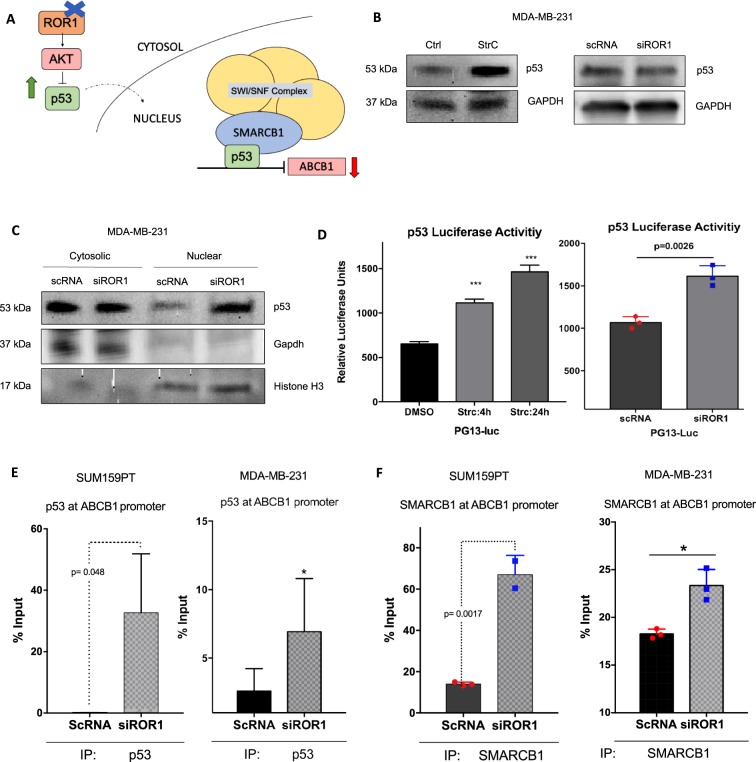
ROR1 promotes ABCB1 transcription by reducing p53/SMARCB1 at ABCB1 promoter. (**A**) P13K/AKT/p53/SMARCB1 regulation of ABCB1 transcription. (**B**) Immunoblot investigating global cell p53 levels after ROR1 siRNA knockdown and inhibition with StrC. Full length blots are presented in the Supplementary Figs. S12 and S13. (**C**) Immunoblots following subcellular fractionation assessing p53 levels in the cytosol and nucleus after ROR1 knockdown. Full length blots are presented in the Supplementary Fig. S14. (**D**) Luciferase reporter assay monitoring p53 activity in MDA-MB-231 after siROR1 and StrC treatment. (**E**,**F**) Chromatin immunoprecipitation assay investigating p53 (**E**) and SMARCB1 (**F**) binding to ABCB1 promoter after ROR1 knockdown. All statistical analyses via student’s t test. N = 3. *p < 0.05, ***p < 0.001.

ABCB1 transcription is regulated by several transcription factors but is most notably repressed by p53^23–25^. p53 levels are tightly regulated by the P13K/AKT growth pathway, which is activated by ROR1 signaling^8,26^ (Fig. 6A). We previously showed inhibition of P13K/AKT after ROR1 modulation^10^. To investigate if ROR1 inhibition modulated p53 levels, we monitored global p53 levels but also its nuclear localization and transcriptional activity. Immunoblots revealed minimal changes in p53 levels after ROR1 knockdown but a significant increase after ROR1 inhibition with Strc (Fig. 6B). We also observed an increase in p53 accumulation in the nucleus after ROR1 knockdown suggesting an increase in its transcriptional activity (Fig. 6C). Using a luciferase reporter system containing p53 responsive elements, we confirmed an increase in p53 transcriptional activity after both ROR1 knockdown and inhibition with Strc (Fig. 6D). Further corroborating these findings, chromatin-immunoprecipitation of p53-bound chromatin, revealed an increase in p53 occupancy of the ABCB1 promoter after ROR1 inhibition (Fig. 6E). These data suggest ROR1 inhibition increases p53 activity, and its repression of ABCB1 transcription. p53 has been reported to often recruit SMARCB1 and the SWI/SNF complex to promoter sequences it regulates^27^. Interestingly, SMARCB1 was previously shown to similarly bind to the ABCB1 promoter and repress transcription^28^. We thus sought to explore the possibility that p53 recruits SMARCB1 to the ABCB1 promoter, exacerbating the ABCB1 transcriptional repression event. Chromatin-immunoprecipitation of SMARCB1-bound chromatin revealed an increase in SMARCB1 occupancy of the ABCB1 promoter after ROR1 inhibition (Fig. 6F). We observed no changes in global levels of SMARCB1 or other SWI/SNF complex members (Supplementary Fig. 5). Altogether, these data suggest ROR1 inhibition promotes p53 occupancy of the ABCB1 promoter, where it recruits SMARCB1 and the SWI/SNF complex for its transcriptional repression activity (Fig. 6A).

### ROR1 inhibition represses doxorubicin efflux from cells

**Figure 7:**
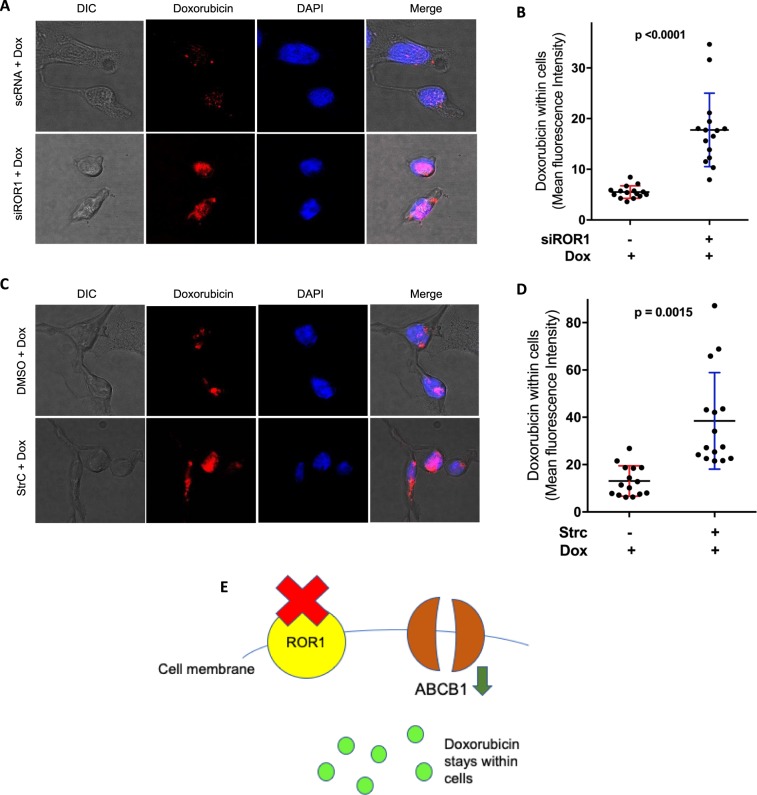
ROR1 inhibition represses drug efflux. (**A**,**C**) Fluorescence confocal microscopy monitoring doxorubicin (red) in MDA-MB-231 nuclei (DAPI/blue) following treatment, after ROR1 knockdown (**A**) or inhibition with StrC (**C**). (**B**,**D**) ImageJ quantification of Doxorubicin within nuclei (mean fluorescence intensity) from images obtained in (**A**,**C**). (**E**) Overall model showing how ROR1 inhibition promotes chemosensitivity by repressing drug efflux from cells. Statistical analyses via student’s t test. N = 3. p < 0.05 considered statistically significant.

To validate our overall findings and to verify if the observed downregulation of ABCB1 expression after ROR1 inhibition correlated with a decrease in chemo drug efflux, we monitored doxorubicin in cells following ROR1 inhibition. Doxorubicin has intrinsic fluorescence^29^ and can be monitored via confocal microscopy. We observed an increase in doxorubicin levels within cells 24 h after treatment, following ROR1 knockdown and Strc inhibition, when compared to control RNA and vehicle treatment groups (Fig. 7, Supplementary Fig. 6). These data suggest ROR1 inhibition represses doxorubicin efflux from cells.

## Discussion

Chemoresistance is the leading cause of therapy failure and high mortality rates in breast cancer^2^. The drug efflux pump ABCB1 plays a key role in promoting chemoresistance by actively effluxing a broad range of chemotherapeutic agents from tumor cells^2,4,5^. Attempts at inhibiting ABCB1 directly have failed clinically due to disruption of normal tissue and lack of specificity^7^. ABCB1 is ubiquitously expressed in non-malignant human tissue making it a poor candidate for targeted therapy^7^. Inhibiting ABCB1 by targeting its cancer-specific upstream regulators thus represents a safer approach, mitigating the damage to normal tissue. So far, few upstream regulators of ABCB1 have been described. Here we propose ROR1 as a clinically-targettable upstream regulator of ABCB1. We demonstrate that inhibiting ROR1 represses ABCB1 protein and mRNA as well as ABCB1 drug-effluxing activity. We also demonstrate that ROR1 inhibition potentiates chemo drug response in breast cancer cells.

ROR1 is an oncofetal tyrosine kinase highly expressed in several neoplasms but minimally expressed in normal adult tissue making it an appealing candidate for targeted therapies^9^. More interestingly, ROR1 was previously shown to be enriched in chemoresistant breast^8^ and ovarian^11^ cancers. High ROR1 expression has also been found to be associated with stemness and tumor recurrence in ovarian cancer, breast cancer, glioblastoma and chronic lymphocytic leukemia^8,11–14^. In concordance with these findings, our analyses suggest ROR1 is highly expressed in patients with poor response to chemo agents, and enriched following chemotherapy.

The ROR1 signaling pathway in breast cancer culminates in apoptosis inhibition, proliferation and metastasis^8,10–12^. Upon binding of its ligand Wnt5a, ROR1 recruits Src, CK1ε and Rab GTPases leading to downstream activation of the MAPK/ERK and P13K/AKT growth pathways^22,30,31^. Both P13K/AKT MAPK/ERK have been shown to be highly activated in chemoresistant malignancies where they promote tumor persistence by inhibiting drug-induced apoptosis^32,33^. Interestingly, activation of the MAPK/ERK pathway also inhibits proteasomal degradation of ABCB1 via downregulation of UBE2R1, a member of the UPS system^20^. Stabilized ABCB1 is then allowed to accumulate and promote chemoresistance via drug effluxing. We observed inhibition of the MAPK/ERK pathway after ROR1 inhibition, which correlated with increased proteasome activity. This presumably led to the reduced levels of ABCB1 protein observed after ROR1 inhibition.

The P13K/AKT pathway, which is activated by ROR1, is a key regulatory pathway for tumor suppressor p53^26^. Phosphorylation of MDM2 by AKT, promotes its nuclear localization where it ubiquitinates p53. Ubiquitinated p53 is then degraded by the UPS system leading to a drop in cellular p53 levels^26^. Corroborating these findings, we observed an increase in p53 levels after inhibition of ROR1 and P13K/AKT. Interestingly, changes in p53 were only minimal after ROR1 knockdown. p53 is rapidly degraded by the proteasome and only drastic changes in p53 accumulation can lead to detectable changes in cellular p53 levels. It is likely that while p53 levels increased after ROR1 knockdown, the change was not remarkable enough to be detected via immunoblotting. P13K/AKT’s repression of p53 has implications for chemoresistance; p53 is a key transcriptional repressor of ABCB1^23–25^. In several instances, inhibitors of the P13K/AKT pathway have been shown to reverse multi-drug resistance by repressing ABCB1, presumably via the AKT/MDM2/p53/ABCB1 axis^5,34^. We observed an increase in p53 nuclear localization and activity after ROR1 and P13K/AKT inhibition which correlated with increased p53 at the ABCB1 promoter. This would explain the observed repression of ABCB1 transcription after ROR1 inhibition. p53 frequently recruits SMARCB1 and members of the SWI/SNF complex to promoter sequences it regulates^27^. Intriguingly, SMARCB1 has also been shown to bind the ABCB1 promoter and repress transcription^28^. We thus sought to assess if the increased levels of p53 at the ABCB1 promoter would correlate with increased levels of SMARCB1. Following ROR1 knockdown, SMARCB1 was significantly increased at the ABCB1 promoter. We thus speculate p53 and SMARCB1 work in tandem to repress ABCB1 transcription following ROR1 inhibition.

Our overall findings suggest ROR1 inhibition could potentiate existing chemotherapy agents by repressing ABCB1 and drug efflux. Unlike ABCB1, ROR1 inhibition has shown promise clinically. UC-961, a humanized monoclonal antibody for ROR1 has shown potency in chronic lymphocytic leukemia, ovarian cancer, B-cell lymphoma and breast cancer^35–37^ (clinical trials: NCT03088878, NCT02860676, NCT02222688, NCT02776917). In these studies, ROR1 inhibition has been shown to synergize with various drug agents for tumor eradication. Because ROR1 is enriched in cancer stem cell populations, ROR1 inhibition would also repress cancer stemness, a key feature of recurrent and chemoresistant neoplasms.

In conclusion, by repressing ABCB1, increasing chemosensitivity and reducing cancer stemness, we speculate inhibition of ROR1, a cancer-specific receptor, in combination with chemotherapy, represents a viable therapeutic strategy for chemoresistant breast cancer and other ROR1-expressing malignancies.

## Methods

### Cell culture

MDA-MB-231 (ATCC HTB 26) were cultured in DMEM/F-12 supplemented with 10% FBS, insulin (10 μg/ml), non-essential amino acids, sodium pyruvate, penicillin (100 U/ml) and streptomycin (0.1 mg/ml). SUM-159PT were cultured in Ham’s F-12 media supplemented with 5% FBS, insulin (5 μg/ml) and hydrocortisone (1 μg/ml). Multidrug resistant SUM-159PT (SUM-159PT/R) were cultured in DMEM/F-12 with 10% FBS containing either 200 nM Paclitaxel or 100 nM Doxorubicin (Dox), in an alternating way. Both naïve and multidrug resistant SUM159PT were a gift from Dr. Raman Dayanidhi at University of Toledo, Ohio, USA. All cells were maintained at 37 °C with 5% CO_2_.

### ROR1 knockdown, overexpression and inhibition

ROR1-siRNA (siROR1) (Thermofisher, AM16708) was transfected into cells via lipofection according to manufacturer’s protocol. Briefly, cells were incubated with siROR1 or a scramble RNA control (scRNA) and Lipofectamine RNAi/Max reagent (Thermofisher, Waltham, MA, USA) in serum-free Opti-MEM (Thermofisher, Waltham, MA, USA) for 24 h. Cells were then rescued in complete growth media for 2 h prior to any further experimentation. ROR1 was inhibited with Strictinin, an ROR1 inhibitor previously described and isolated in our laboratory (10). ROR1 was overexpressed in MCF-7 using a pCDNA-3.1-ROR1 construct (NM_001083592.1, GenScript, Piscataway, NJ, USA) encoding an ROR1 ORF according to manufacturer’s protocol.

### MTT assays

MTT cell viability assays (Thermofisher, Waltham, MA, USA) according to the manufacturer’s protocol, as previously reported (10). Cells were seeded in a 96-well plate at a density of 25,000 cells/well. Following knockdown and/or treatments, media was replaced with fresh media. MTT dye was added and incubated at 37 °C for 2 h. DMSO was added to solubilize the formazan and absorbance was read at 540 nm. Blank wells were included and non-specific absorbance subtracted from final absorbance readings. Cell viability was computed as percentage of vehicle treatment or control RNA control.

### Apoptosis assay

Apoptosis was assessed via Annexin V staining using the CellMeter Phosphatidylserine Apoptosis Assay Kit (22793, AAT Bioquest, Sunnyvale, CA), according to manufacturer’s instructions. Briefly, cells were plated in 96-well plates at a density of 80,000 cells/well. Following StrC and/or Dox/Cis treatment, cells were stained with a fluorescently-tagged Annexin-V dye. Fluorescence intensity, representative of apoptosis, was monitored using a Tecan microplate reader. Strictinin was isolated as per the method we previously described^10,38^.

### Immunofluorescence

Following knockdown and/or treatments, cells were plated at a density of 15,000 cells/well in wells of Lab-Tek chamber slides (Sigma-Aldrich, St. Louis, MO, USA) and fixed in pre-chilled methanol for 5 minutes at −20 °C. Cells were washed with PBS and incubated with blocking buffer (5% BSA/PBS) at room temperature for one hour. The cells were then incubated at 4 °C with γH2AX primary antibody (#2577 S, Cell signaling, Danvers, MA, USA) diluted 1:400 in blocking buffer on a shaker overnight. Following primary antibody incubation and PBS washes, cells were incubated with Alexa-fluor-647 secondary antibody (Thermofisher, Waltham, MA, USA) in blocking buffer at room temperature for an hour. For ABCB1 staining, an ABCB1/MDR-1 mouse primary (D-11, Santa Cruz Biotechnology, Dallas, TX, USA) and a Cy5 affinipure anti-mouse secondary (Jackson Immunoresearch Labs, West Grove, PA, USA) were used. The cells were then washed again with PBS and incubated with a DAPI stain solution (1 µg/ml in PBS) for 5 minutes at room temperature. After additional washing steps, cells were imaged on a Leica DMi8 Microscope (Leica, Wetzlar, Germany). γH2AX foci were analyzed and quantified using ImageJ (NIH, Bethesda, MD, USA). For Doxorubicin efflux experiments, mean fluorescence intensity was computed using NIS-Elements (Nikon Instruments, Melville, NY, USA).

### Protein isolation and quantification

Following knockdown and/or treatments, cells were scraped and centrifuged to obtain a cell pellet. To obtain whole cell lysates, cells were lysed with RIPA buffer (150 mM NaCl, 1.0% Triton-X-100, 0.5% Sodium deoxycholate, 0.1% SDS, 50 mM Tris, pH 8.0) with 1 mM PMSF. Following an ice incubation of 15 minutes, the solution was centrifuged for 15 minutes and the protein supernatant was recovered. For experiments requiring nuclear and cytosolic proteins isolated separately, subcellular fractionation was performed. To obtain the cytosolic fraction, cells were resuspended in a buffer containing 10 mM HEPES, 1.5 mM MgCl_2_, 10 mM KCl, 0.5 mM DTT, 0.05% NP-40 (pH 7.9) on ice for 10 minutes then centrifuged at 3000 rpm for 10 minutes and the supernatant collected. For nuclear proteins, the ensuing pellet was homogenized in a buffer containing 5 mM HEPES, 1.5 mM MgCl_2_, 0.2 mM EDTA, 0.5 mM DTT, 26% glycerol (v/v), 300 mM NaCl (pH 7.9) using a syringe and incubated on ice for 30 minutes. Cell homogene was then centrifuged at 24,000 g for 20 minutes at 4 °C and the nuclear protein lysate supernatant collected. Protein quantification for all samples was identified using the Bradford Assay using Pierce 660 nm Protein Assay reagent (ThermoFisher, Waltham, MA, USA).

### Immunoblots

Protein samples were boiled in 2x Laemmli Buffer and 355 mM β-mercaptoethanol (Millipore Sigma, ES-007-E). Following SDS-PAGE, the proteins were transferred to a nitrocellulose membrane, which was blocked in 5% non-fat dry milk/TBS-T(20 mM Tris, 150 mM NaCl, 0.1% Tween 20) and then washed in TBS-T. Primary antibody incubations were done according to supplier recommendations, overnight at 4 °C. Secondary HRP-linked anti-mouse or anti-rabbit (Cell Signaling, Danvers, MA, USA) antibody incubations were done at room temperature for 1 hour prior to TBS-T washes. Protein bands were imaged via enhanced chemiluminescence. 1° Antibodies: ROR1 (Cell Signal, #D6T8C), GAPDH (Cell Signal, #D16H11), p-ERK (Cell Signal, #9101), p-MEK1/2-ser217/221 (Cell Signal, 9121), p-RSK1 (Cell Signal, 9344 S), ABCB1 (Cell Signal 12683 S), p53 (Cell Signal, #9282 T), Histone H3 (ab12079, Abcam, Cambridge, MA, USA,), MEK1 (Cell Signal, 4694 S), ERK (Cell Signal, #9282 T).

### Real-time Quantitative PCR

RNA was isolated from cells using Trizol Reagent (Thermofisher, Cat. No. 15596026) according to manufacturer’s protocol. cDNA synthesis reaction was performed using SuperScript™ III First-Strand Synthesis System (Thermofisher 18080051). Real time quantitative PCR of target sequences was performed using the CFX384 Touch Real-Time PCR Detection System (Bio-Rad) with the following primers: (5′ > 3′) ROR1 (Fwd-TGTTTGTCAAGTTTGGCCCCC/Rev-AGGGAAGGAATGGCGAACTG), ABCB1 (Fwd-ATGGCTACATGAGAGCGGAGG/Rev-GTTGCACCTCTCTGGTCCCC).

### 20S proteasome activity assay

Proteasome activity was assessed using the 20S Proteasome Assay Kit (10008041, Cayman Chemical, Ann Arbor, MI, USA) according to the manufacturer’s protocol. Briefly, following knockdown, cells were lysed and assessed for proteasomal activity using a 20S specific substrate SUC-LLVY-AMC. Upon cleaving of SUC-LLVY-AMC by the proteasome, highly fluorescent products are generated which can be monitored by excitation and emission wavelengths 360 nm and 480 nm, respectively.

### p53 activity luciferase assay

p53 activity was assessed using a PG13-luc (Addgene, #16442) luciferase reporter. The luciferase reporter system contains 13 copies of a p53-binding consensus sequence. Cells were transfected with the PG13-luc reporter in 6-well dishes prior to treatment and/or knockdown.

Luciferase activity was measured using the Dual-Glo Luciferase Assay System reagent (Promega, E2920).

### Chromatin-immunoprecipitation

p53- or SMARCB1-bound chromatin was immunoprecipitated using Thermofisher’s Pierce Agarose ChIP Kit (Thermofisher, #26156). Briefly, cells were plated in 6-well plates (320,000 cells/well) and transfected and/or treated. Following fixation with 1% paraformaldehyde, cells were lysed and nuclear chromatin-bound protein was isolated. Immunoprecipitation was performed overnight at 4 °C using ChIP-grade p53 (Cell Signal, #9282 T) or SMARCB1 (Cell Signal, #91735) antibodies. qPCR of ABCB1 was performed using these primers ABCB1 (Fwd-ATGGCTACATGAGAGCGGAGG/Rev-GTTGCACCTCTCTGGTCCCC). ABCB1 levels were computed using percent input method.

### Statistical analyses

All data were represented as mean ± standard deviation unless otherwise specified. Analyses of statistical significant differences between groups were conducted using unpaired two-tailed student’s t-test, unless otherwise indicated. P-values less than 0.05 were considered statistically significant. All statistical analyses were performed using GraphPad Prism 7.0 (GraphPad Software Inc, San Diego, CA, USA).

### Patient data analysis

For gene expression of ABCB1 and ROR1 in patients with HER2-negative breast cancer, gene expression data was obtained from GEO (accession code: GSE87455) and analyzed with GEO2R (16). Patients are divided into ROR1-low and -high groups using median expression of ROR1 as cutoff.

## Supplementary information

Supplementary information.

## Data Availability

All data available upon request.
