# Supplementary material for: ROR1 regulates chemoresistance in Breast Cancer via modulation of drug efflux pump ABCB1
Source: Sci Rep. 2020 Feb 4;10:1821. doi: 10.1038/s41598-020-58864-0 (PMC7000766; doi:10.1038/s41598-020-58864-0)
Supplement: Supplementary file 1 — Supplementary information. [file 41598_2020_58864_MOESM1_ESM.pdf]

ROR1 regulates chemoresistance in Breast Cancer via modulation of drug efflux pump ABCB1

Norman Fultang[1], Abhinav Illendula[1], Jianhuang Lin[2], Manoj K. Pandey[3], Zachary Klase[1], Bela Peethambaran[1,*].

[1] Department of Biological Sciences, University of the Sciences in Philadelphia, Philadelphia, Pennsylvania, 19104 United States of America
[2] The Wistar Institute, Philadelphia, Pennsylvania, 19104, United States of America
[3] Department of Biomedical Sciences, Cooper Medical School of Rowan University, Camden, New Jersey, 08103, USA

* Corresponding author: Bela Peethambaran, PhD:
b.peethambaran@usciences.edu

Figure S1

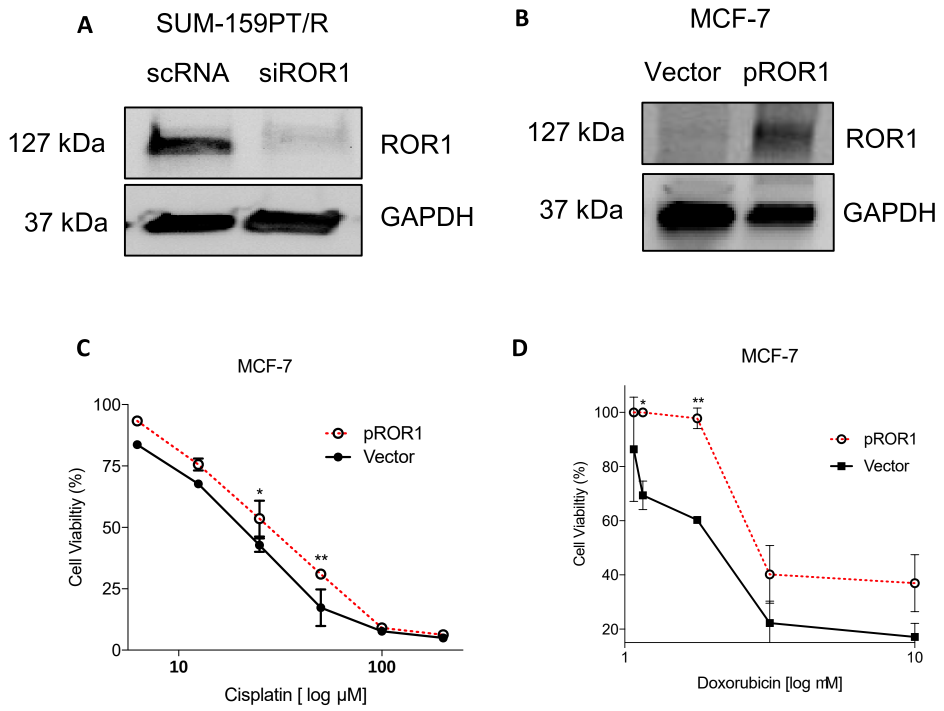
A
SUM-159PT/R
scRNA
siROR1
127 kDa
ROR1
37 kDa
GAPDH
B
MCF-7
Vector
pROR1
127 kDa
ROR1
37 kDa
GAPDH
C
MCF-7
pROR1
Vector
Cell Viability (%)
Cisplatin [log µM]
D
MCF-7
pROR1
Vector
Cell Viability (%)
Doxorubicin [log nM]

Figure S2

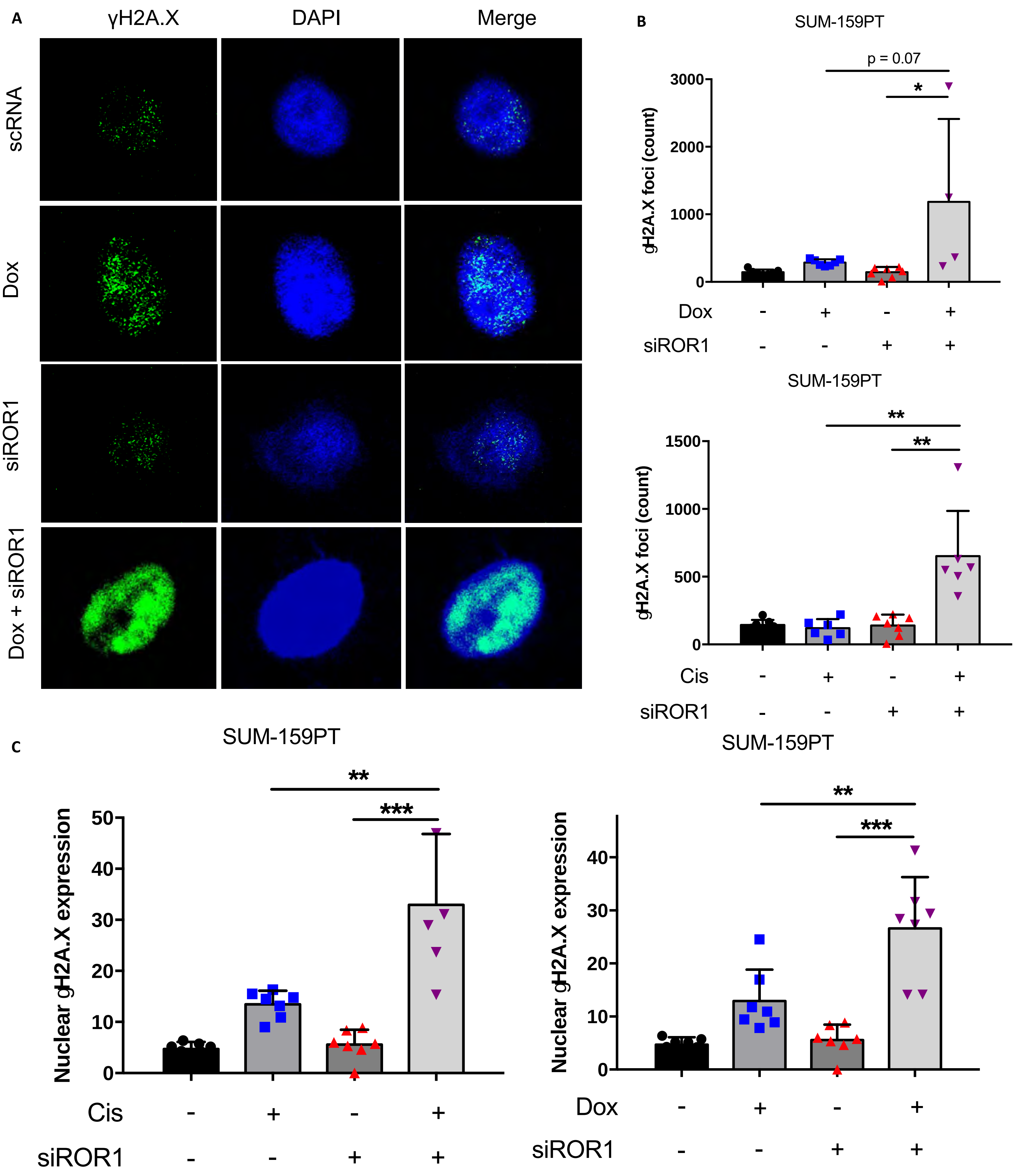
A
γH2A.X
DAPI
Merge
scRNA
Dox
siROR1
Dox + siROR1
B
SUM-159PT
p = 0.07
*
gH2A.X foci (count)
3000
2000
1000
0
Dox - + - +
siROR1 - - + +
SUM-159PT
**
**
gH2A.X foci (count)
1500
1000
500
0
Cis - + - +
siROR1 - - + +
C
SUM-159PT
**
***
Nuclear gH2A.X expression
50
40
30
20
10
0
Cis - + - +
siROR1 - - + +
SUM-159PT
**
***
Nuclear gH2A.X expression
40
30
20
10
0
Dox - + - +
siROR1 - - + +

Figure S3

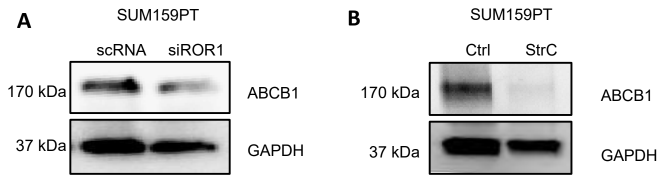
A
SUM159PT
scRNA
siROR1
170 kDa
ABCB1
37 kDa
GAPDH
B
SUM159PT
Ctrl
StrC
170 kDa
ABCB1
37 kDa
GAPDH

Figure S4

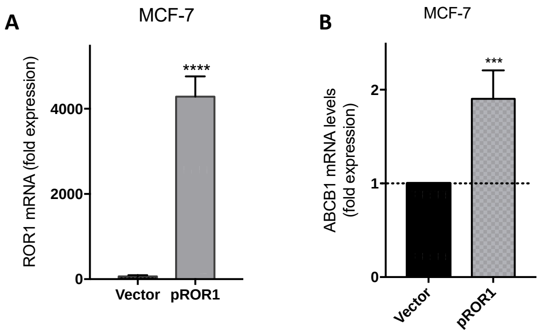

A
MCF-7
ROR1 mRNA (fold expression)
4000
2000
0
****
Vector
pROR1
B
MCF-7
ABCB1 mRNA levels
(fold expression)
2
1
0
***
Vector
pROR1

Figure S5

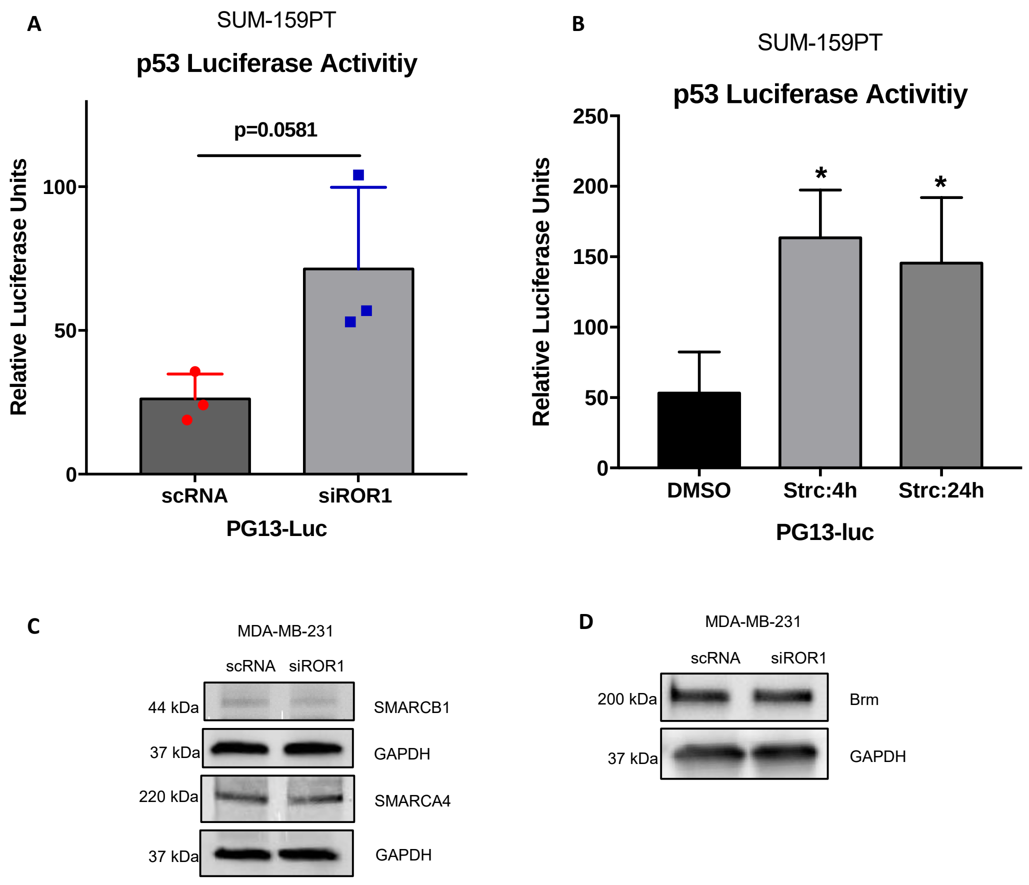
A
SUM-159PT
p53 Luciferase Activitiy
p=0.0581
Relative Luciferase Units
100
50
0
scRNA
siROR1
PG13-Luc
B
SUM-159PT
p53 Luciferase Activitiy
250
200
150
100
50
0
Relative Luciferase Units
*
*
DMSO
Strc:4h
Strc:24h
PG13-luc
C
MDA-MB-231
scRNA
siROR1
44 kDa
SMARCB1
37 kDa
GAPDH
220 kDa
SMARCA4
37 kDa
GAPDH
D
MDA-MB-231
scRNA
siROR1
200 kDa
Brm
37 kDa
GAPDH

Figure S6

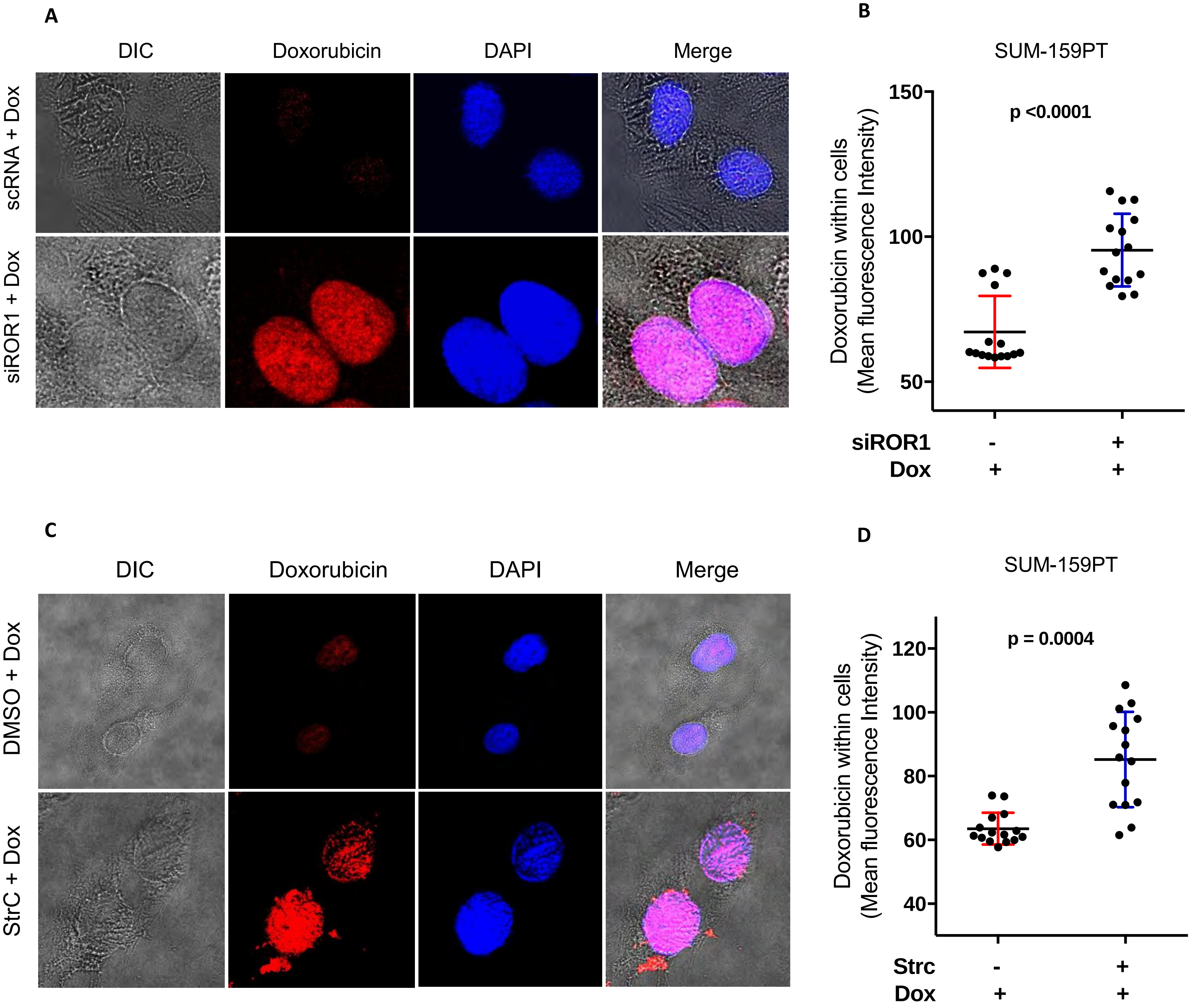
A
DIC
Doxorubicin
DAPI
Merge
scRNA + Dox
siROR1 + Dox
B
SUM-159PT
p <0.0001
150
100
50
Doxorubicin within cells
(Mean fluorescence Intensity)
siROR1
-
+
Dox
+
+
C
DIC
Doxorubicin
DAPI
Merge
DMSO + Dox
StrC + Dox
D
SUM-159PT
p = 0.0004
120
100
80
60
40
Doxorubicin within cells
(Mean fluorescence Intensity)
Strc
-
+
Dox
+
+

Supporting gel data fig 1D

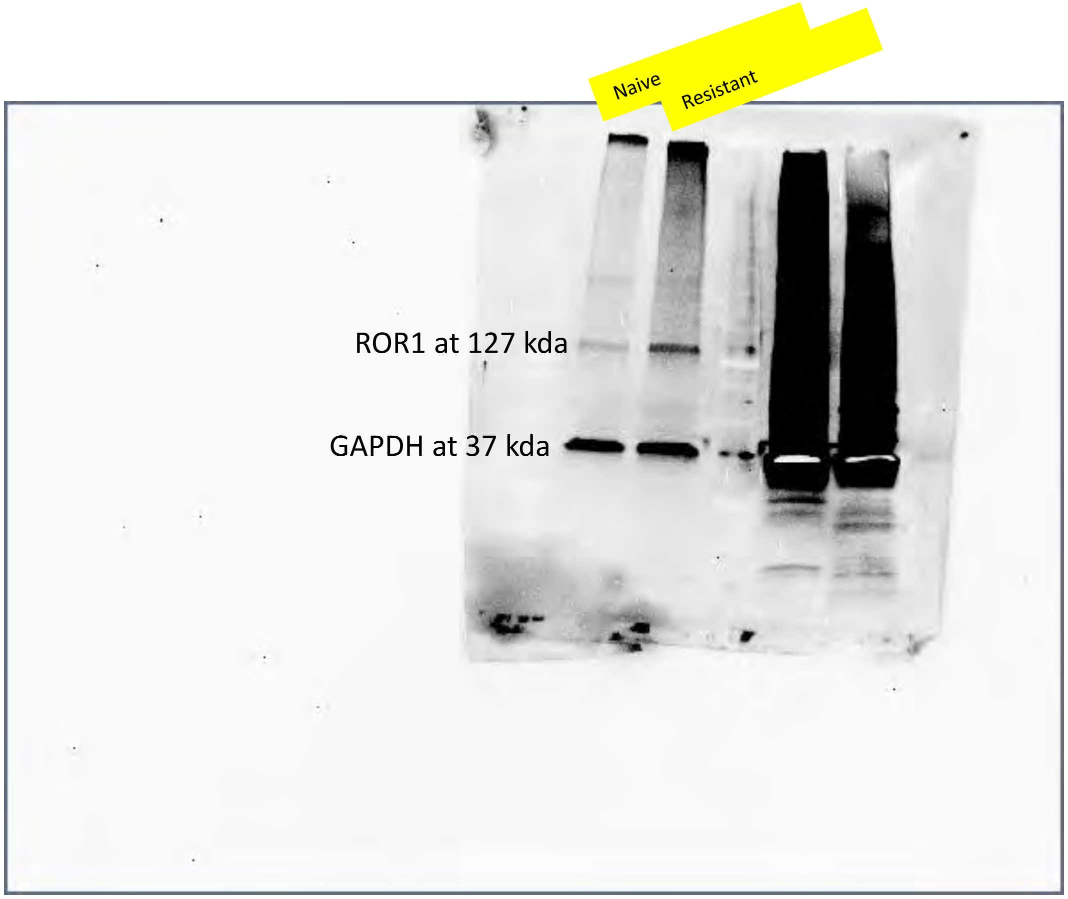

Supporting gel data fig 2A

Figure 2a.

MDA-MB-231

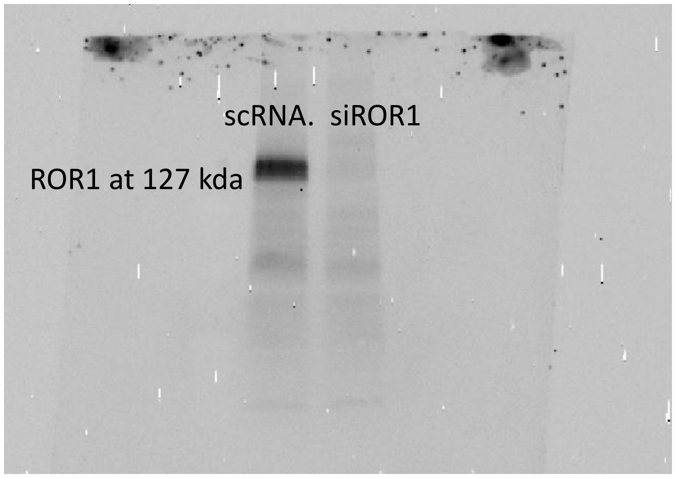

MDA-MB-231

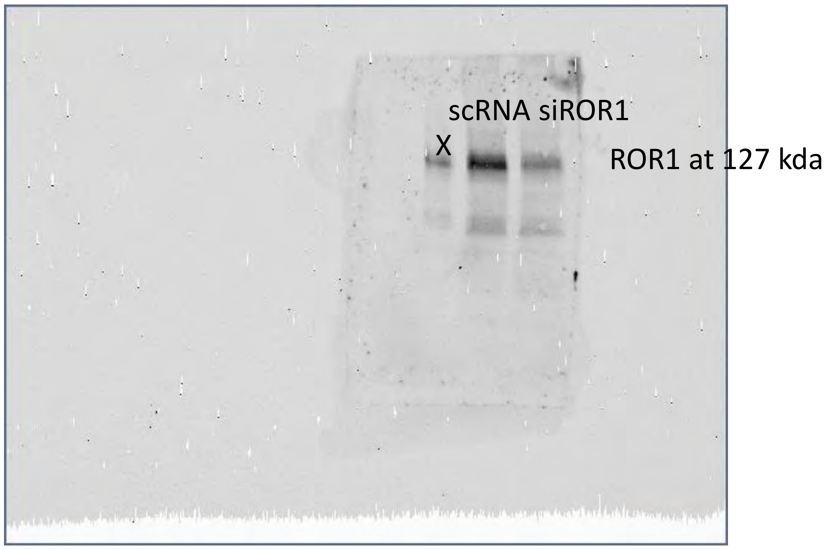

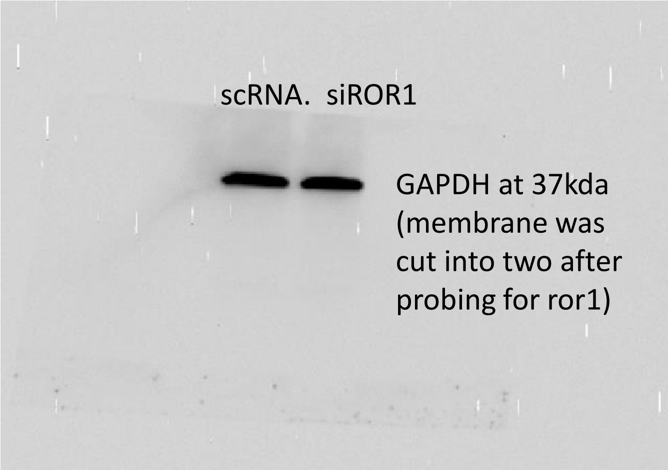

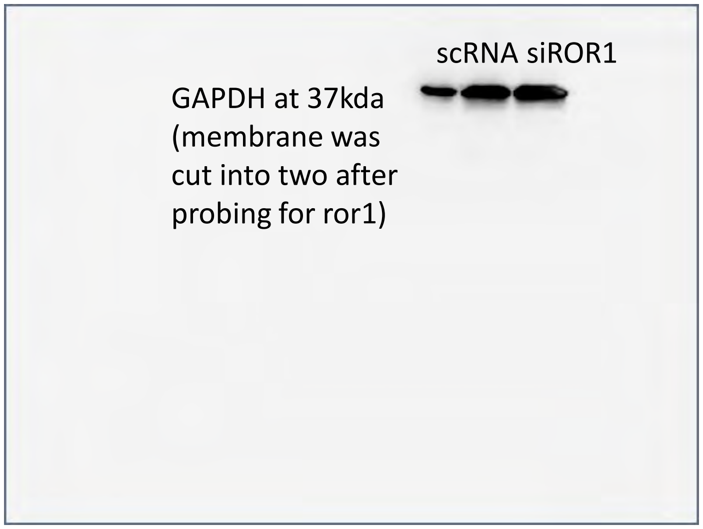

Supporting gel data figure 4A

Figure S9

First panel fig. 4a

Ran on same gel used as first panel of fig 2a.

MDA-MB-231

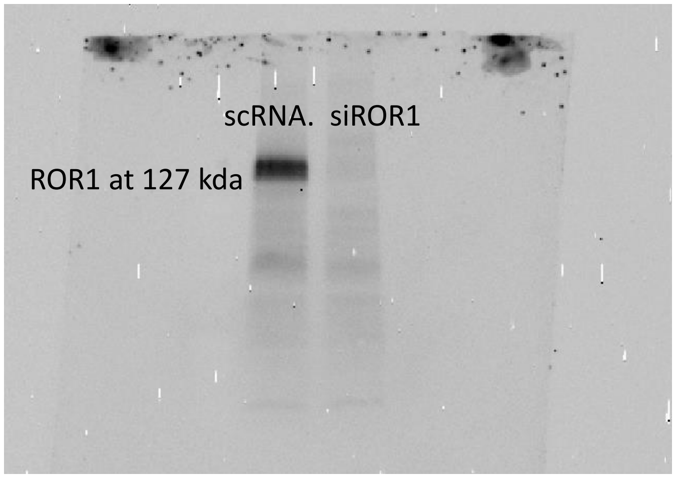

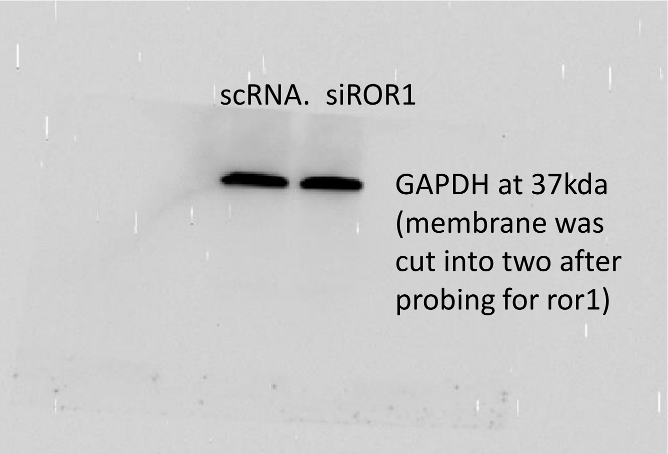

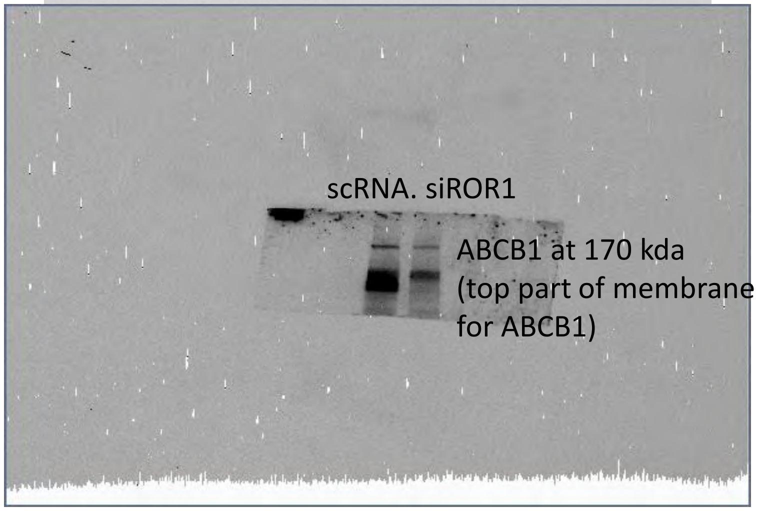

Second panel fig. 4a

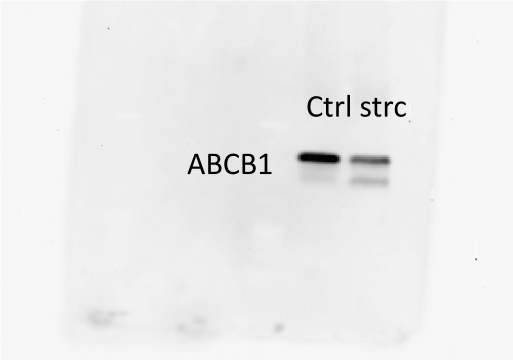

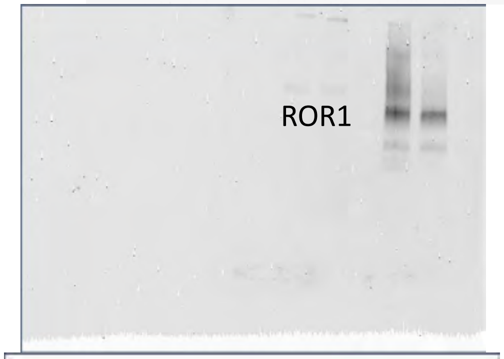

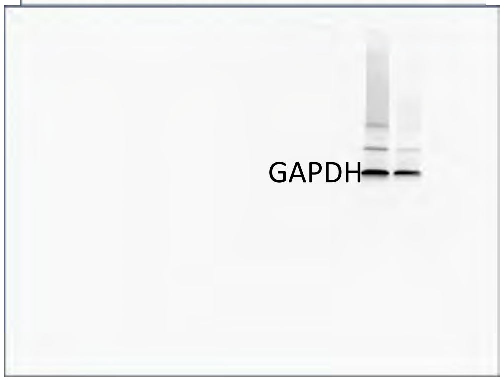

Supporting gel data figure 5B

Figure S10

1 First, we probed whole ERK

ERK

X = Lanes not used

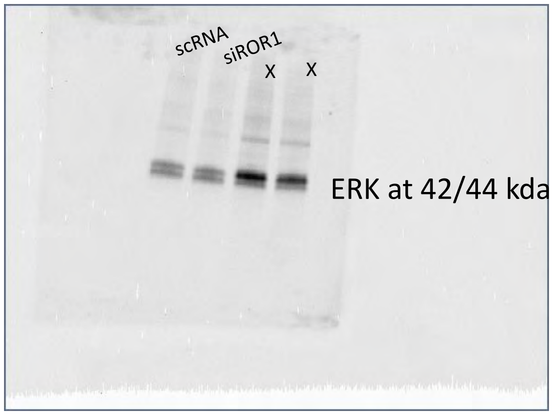

2 We then cut the membrane in between lanes 2 and 3, and stripped it using a harsh stripping buffer (abcam: https://www.abcam.com/ps/pdf/protocols/stripping%20for%20reprobing.pdf)

2 We then probed p-MEK

P-MEK

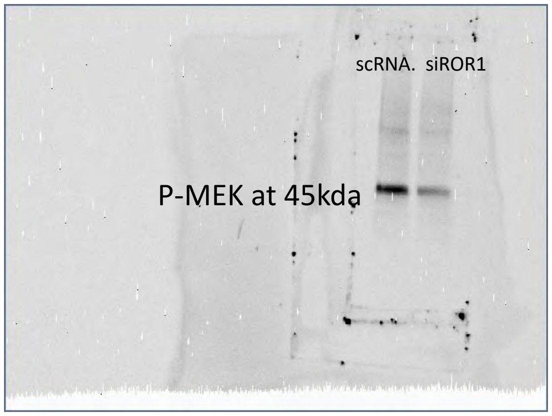

Then GAPDH

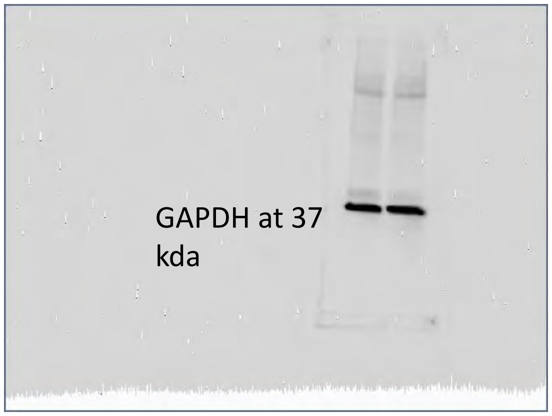

3 We then stripped the membrane one more time and probed p-ERK

P-ERK

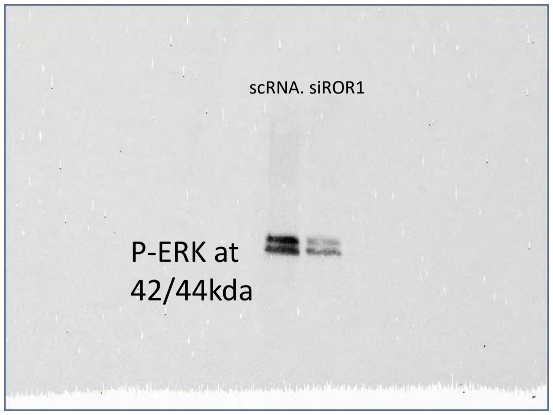

Supporting gel data figure 5B continued

Figure S11

This was run on a new membrane

Second panel fig. 4a

P-RSK1

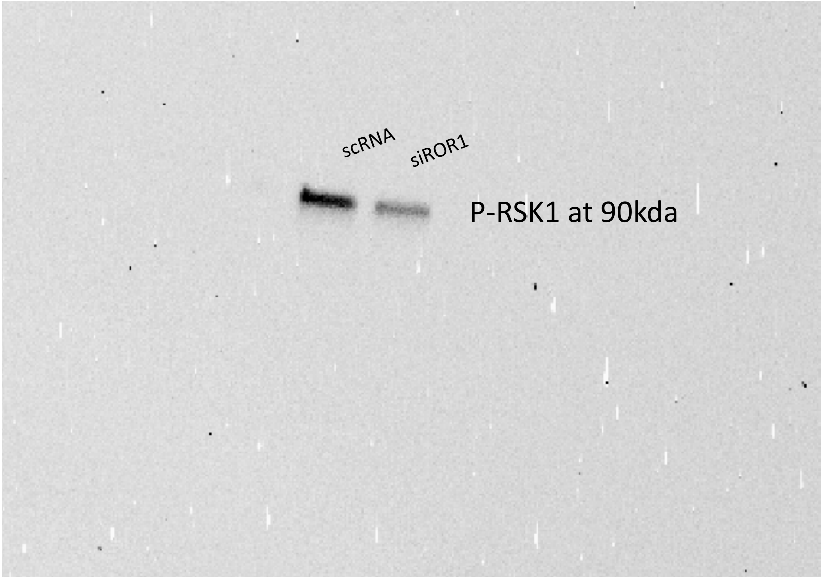

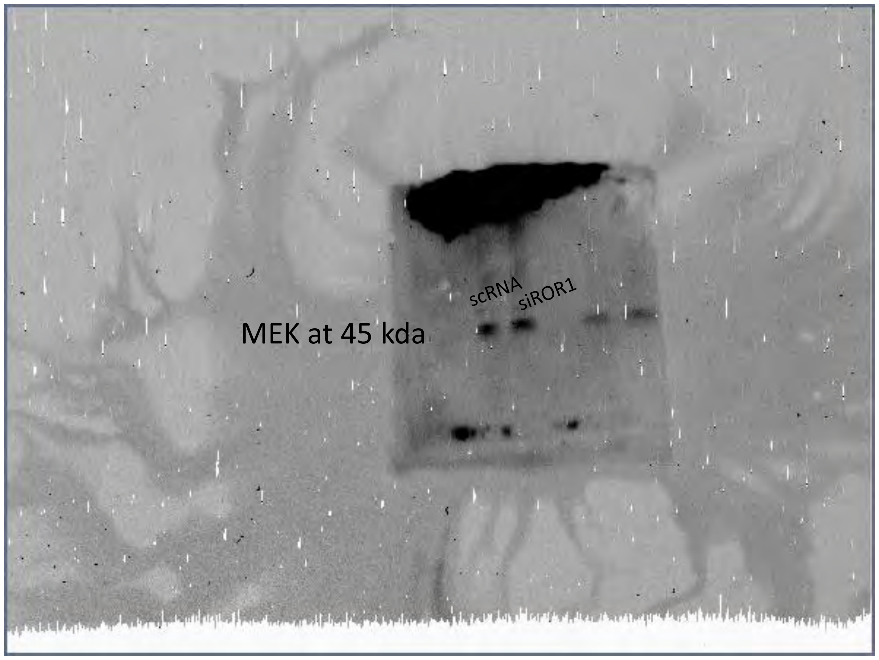

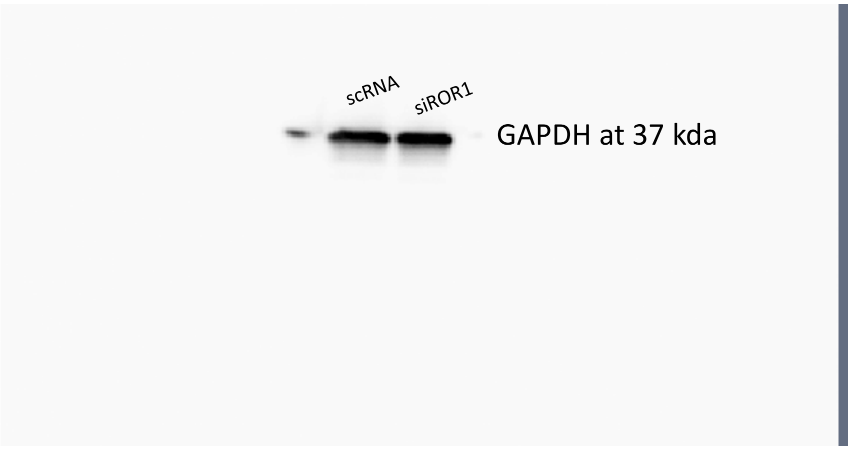

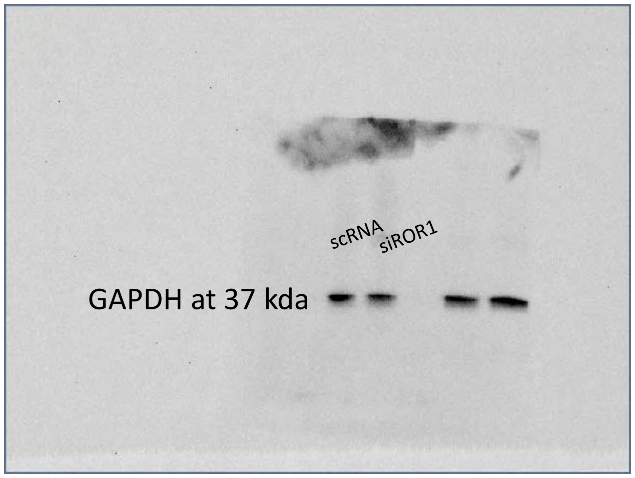

Supporting gel data figure 6B

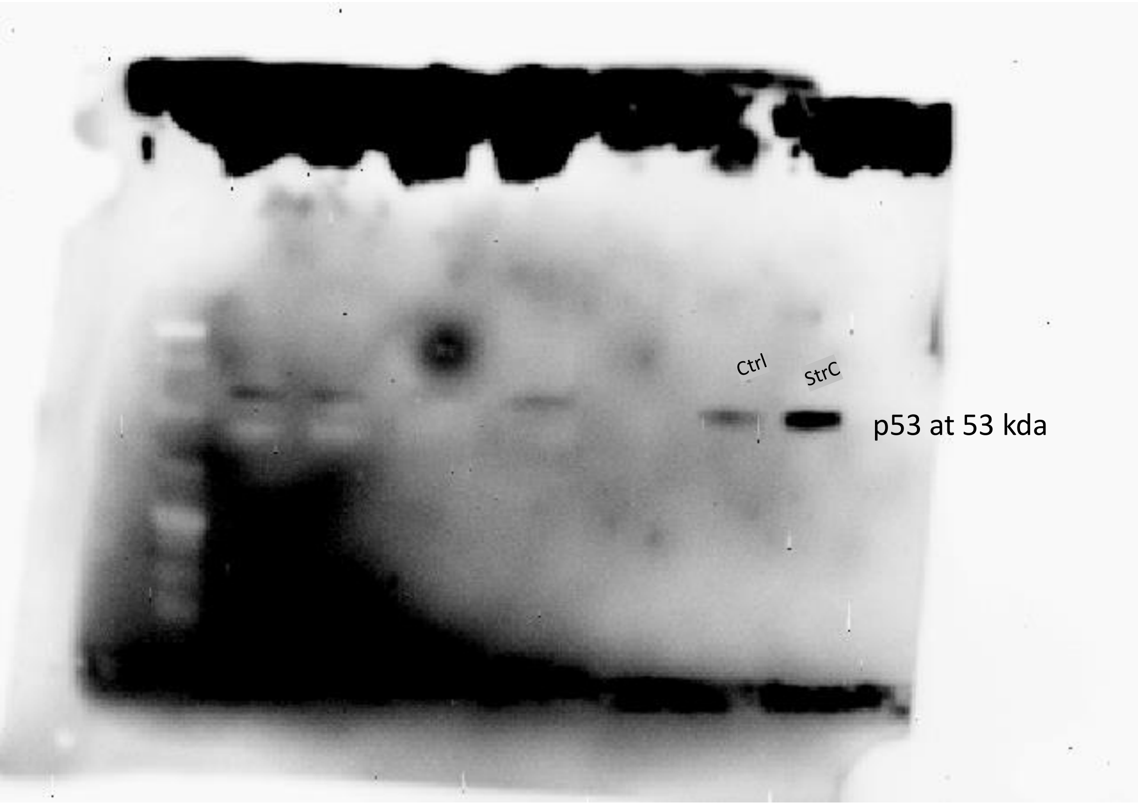

p53 at 53 kda

Membrane was then cut cross at around 50 kda

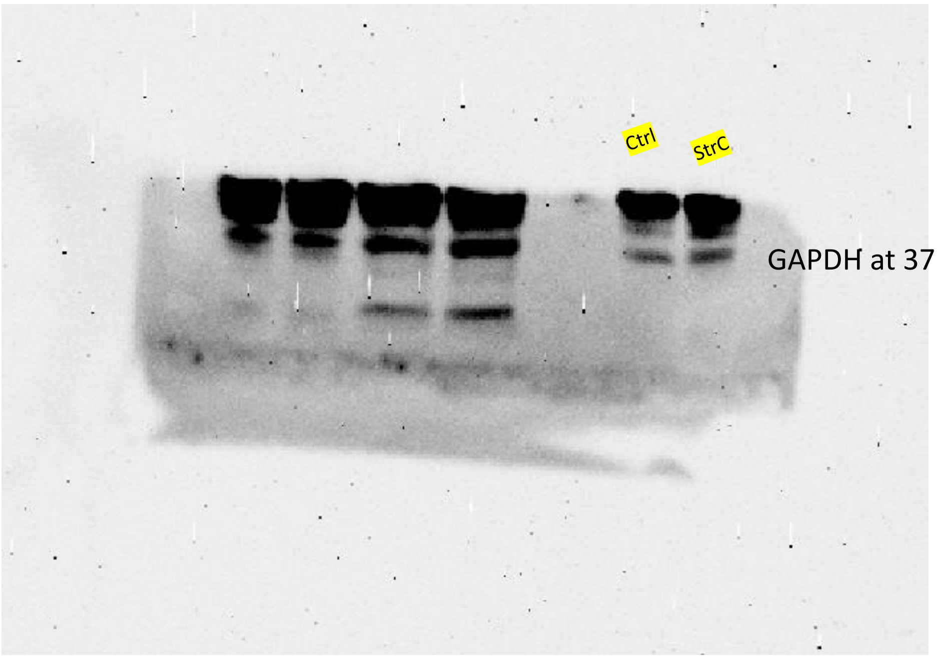

GAPDH at 37 kda

Supporting gel data figure 6B continued

Figure S13

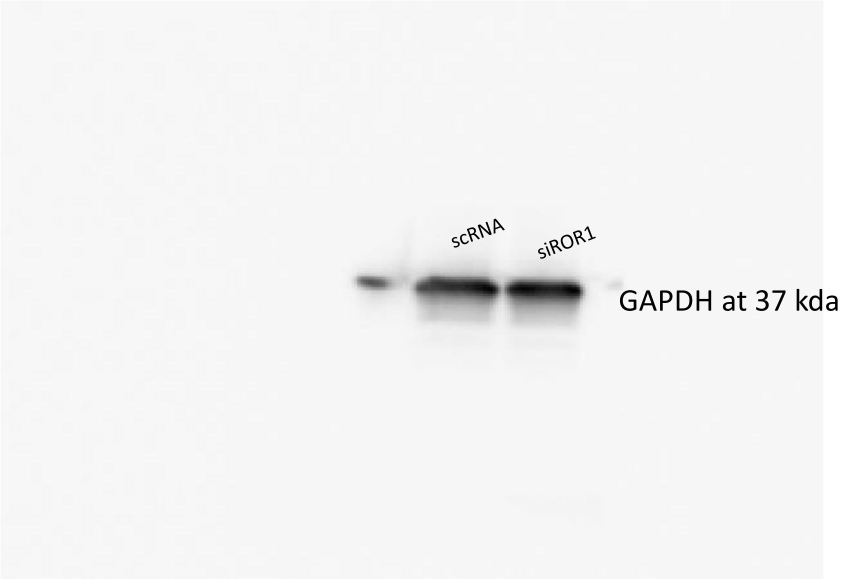

Membrane was then cut cross at around 60 kda

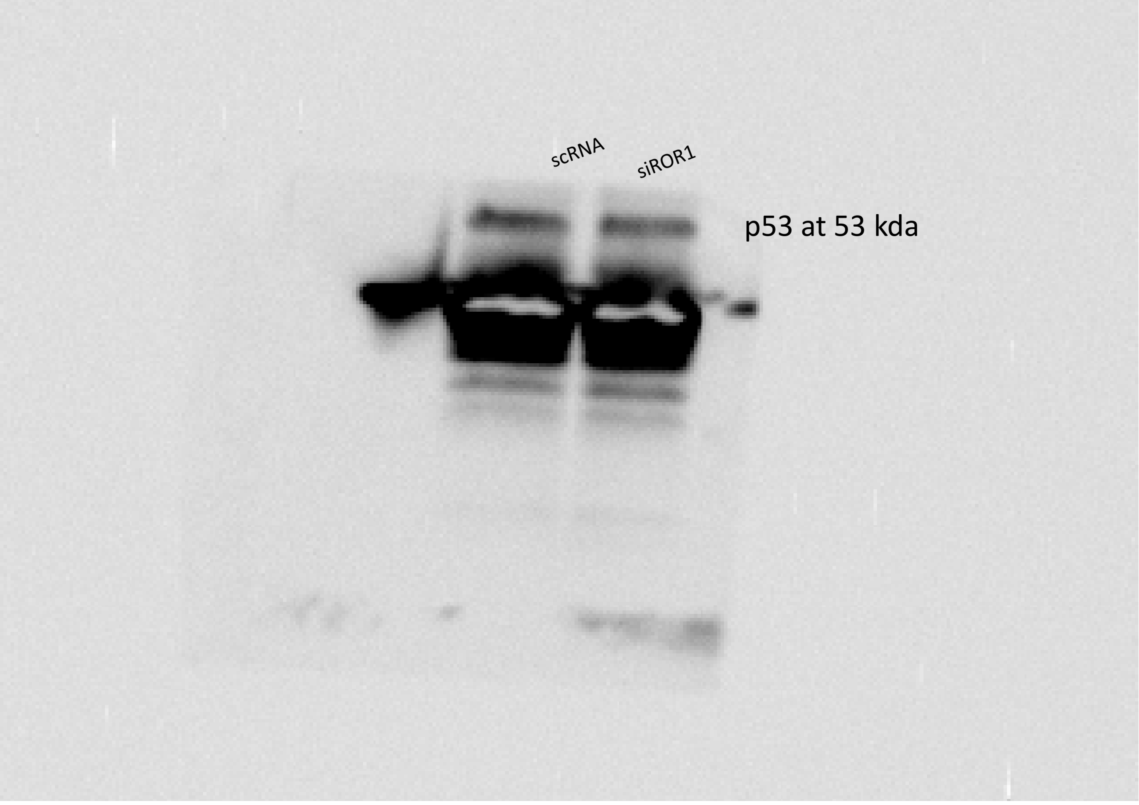

Figure S14

Supporting gel data figure 6C

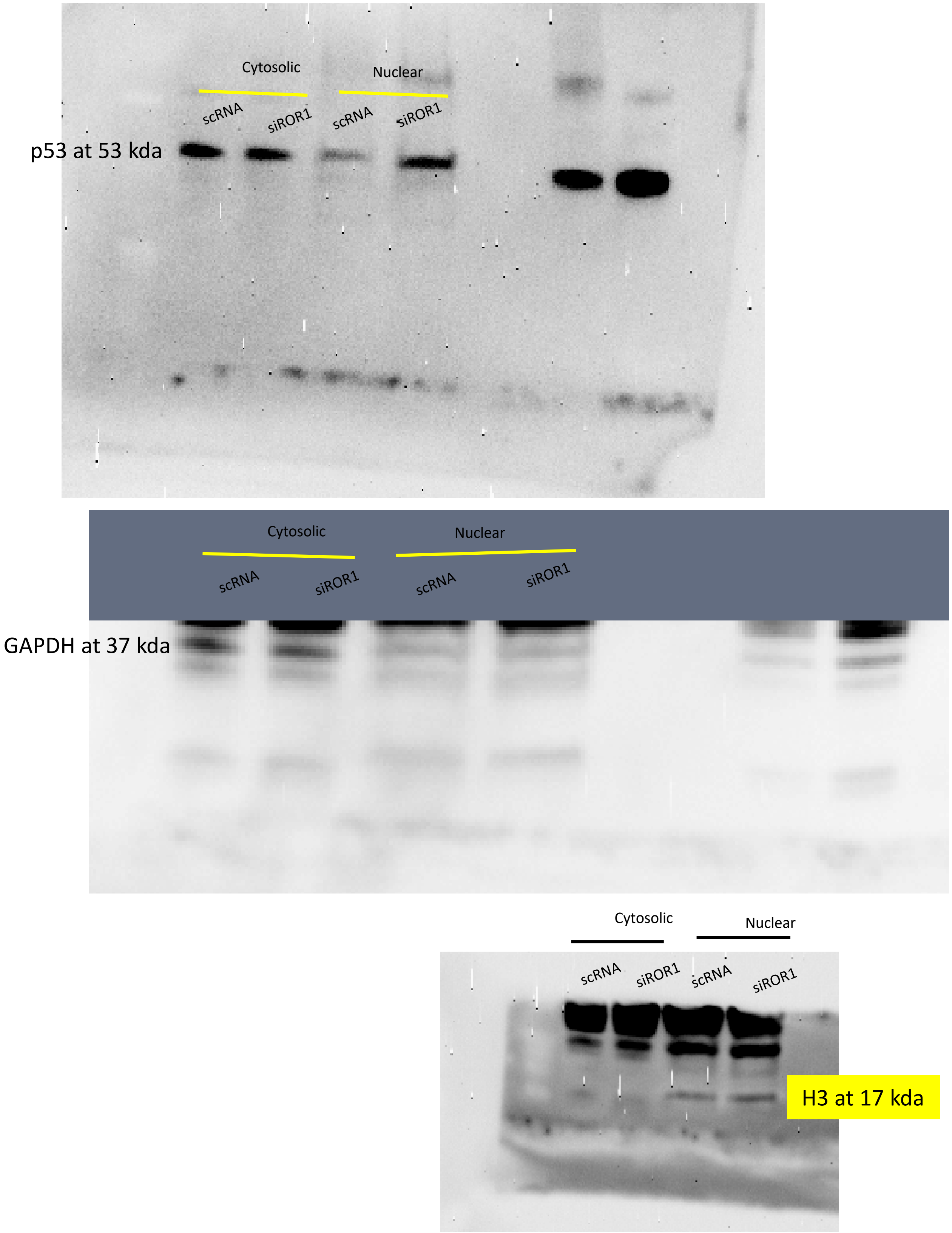

Figure S15

Supporting gel data figure S1A

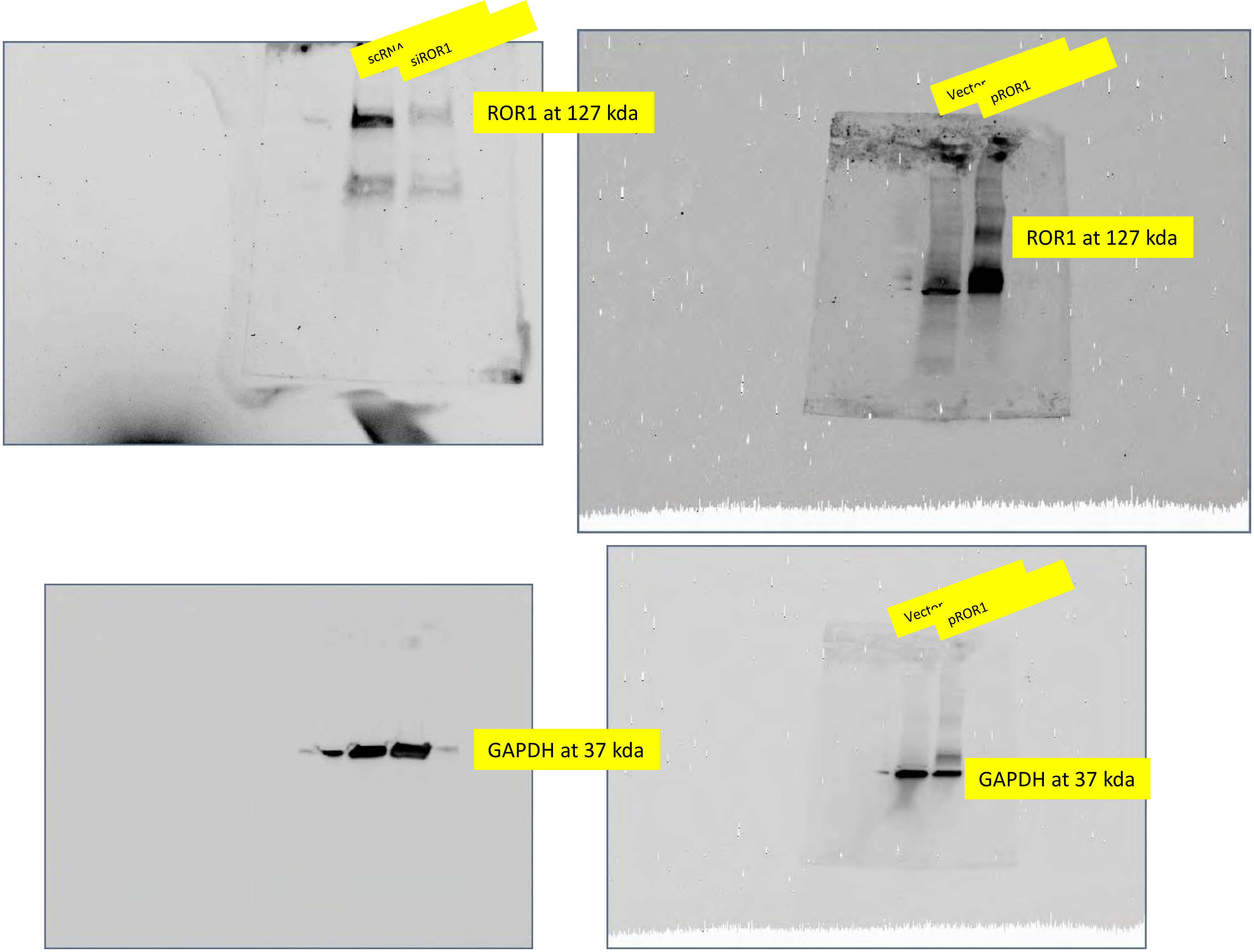

Figure S16

Supporting gel data figure S3A

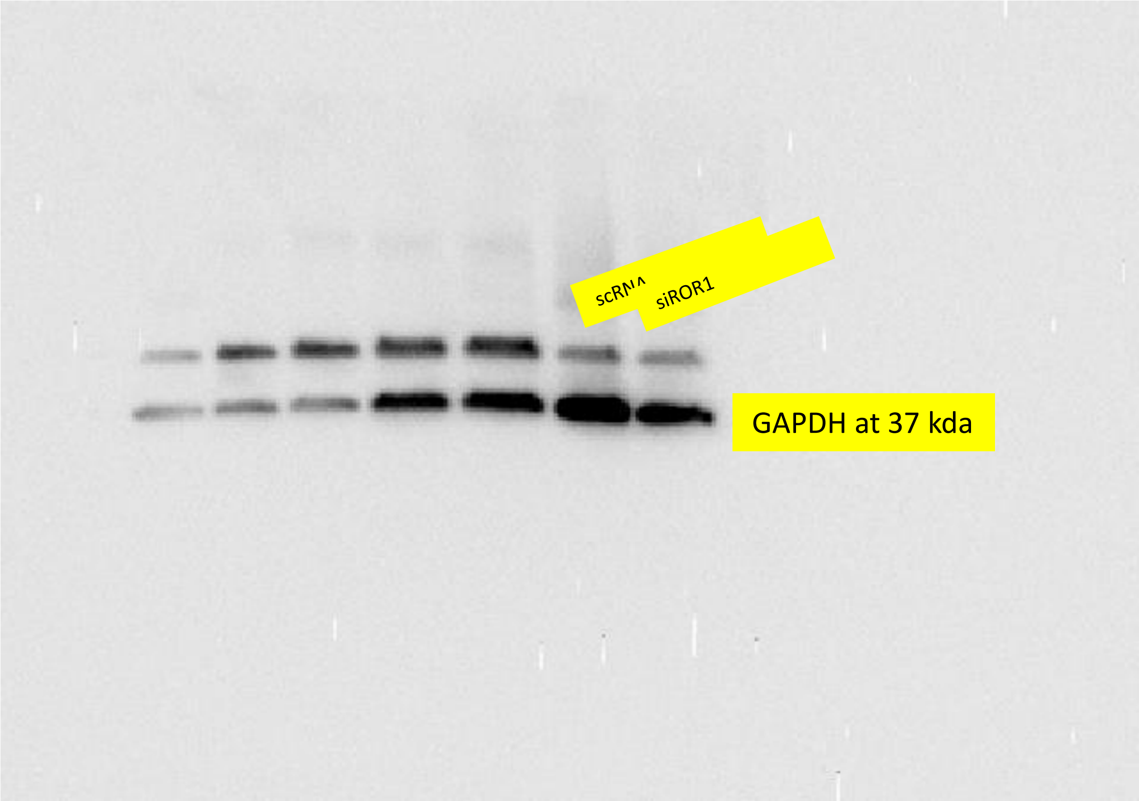

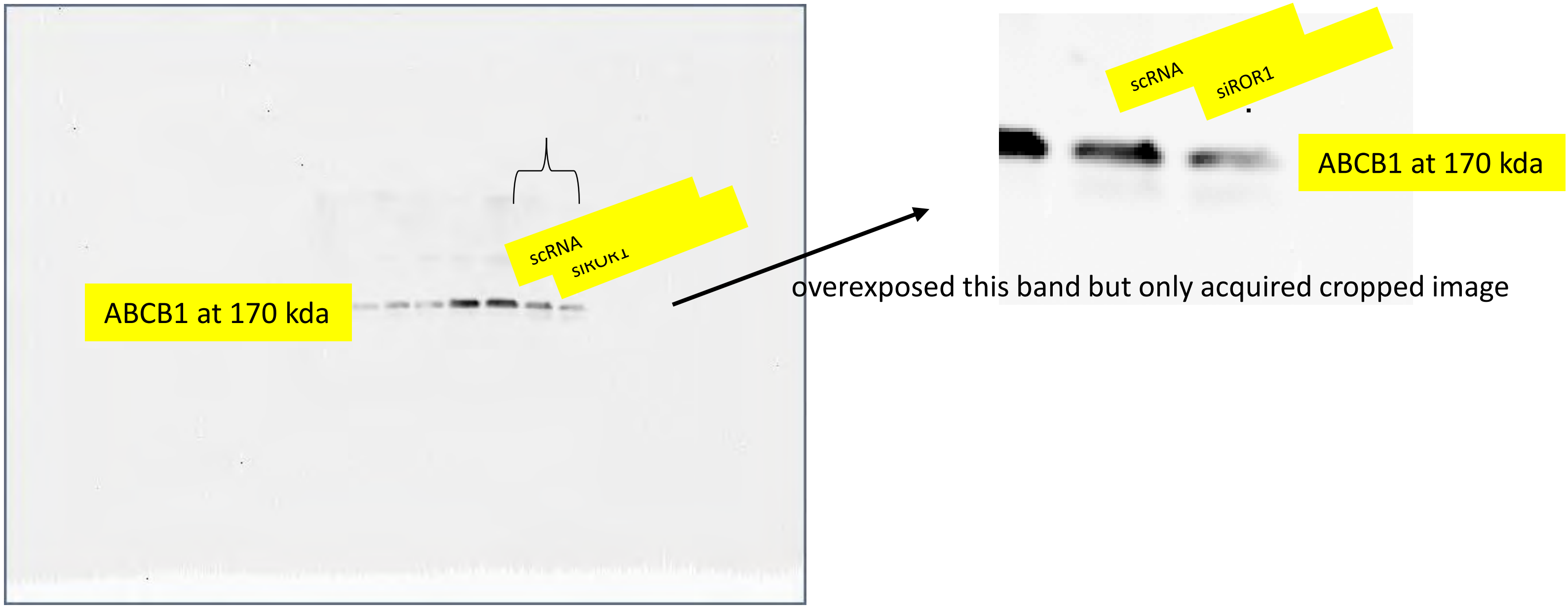

Supporting gel data figure S3B

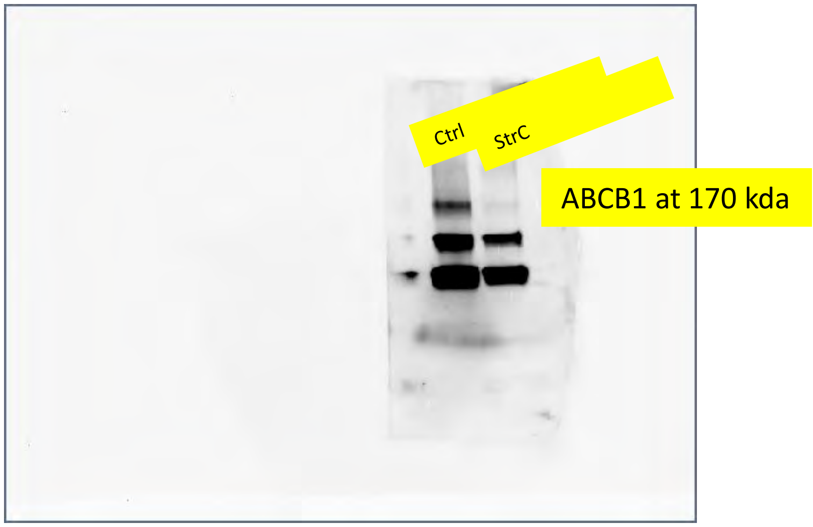

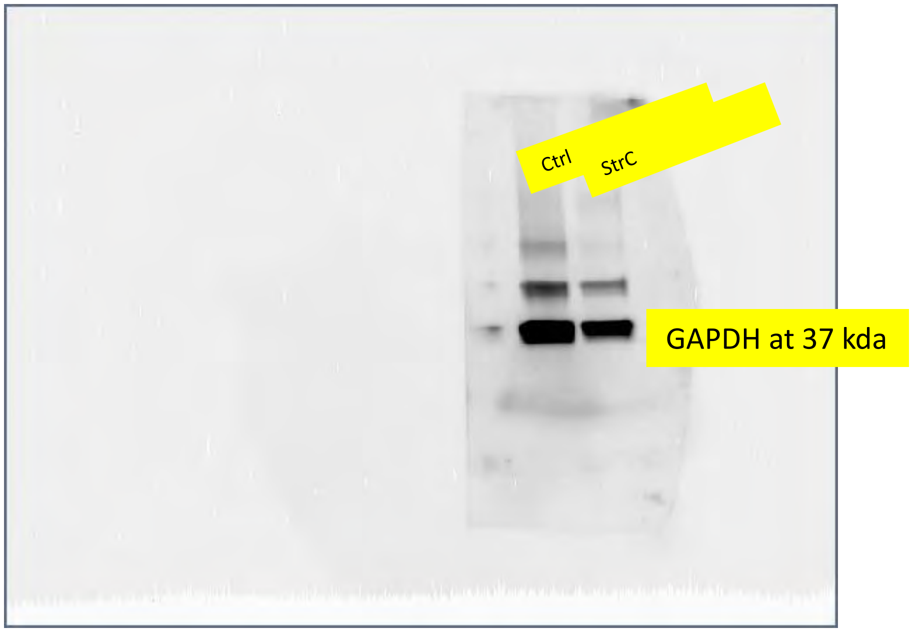

Figure S18

Supporting gel data figure S5A

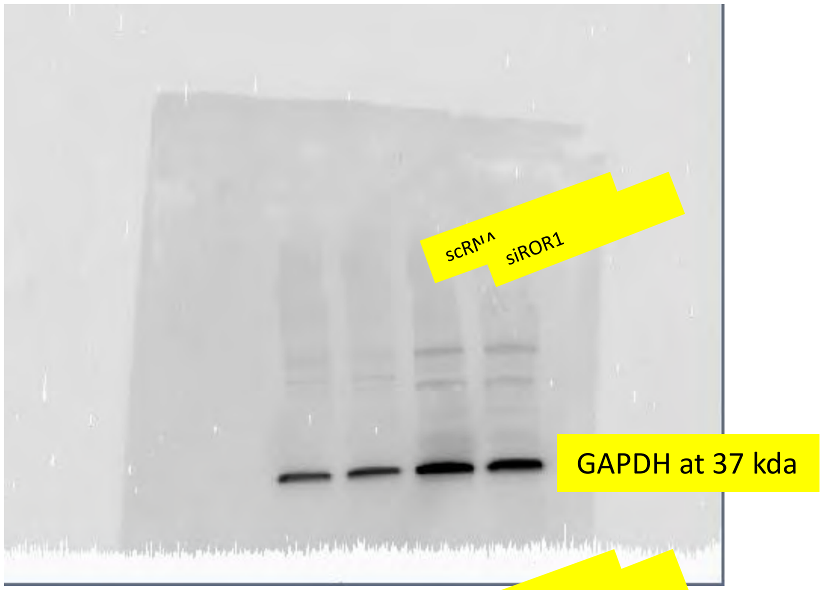

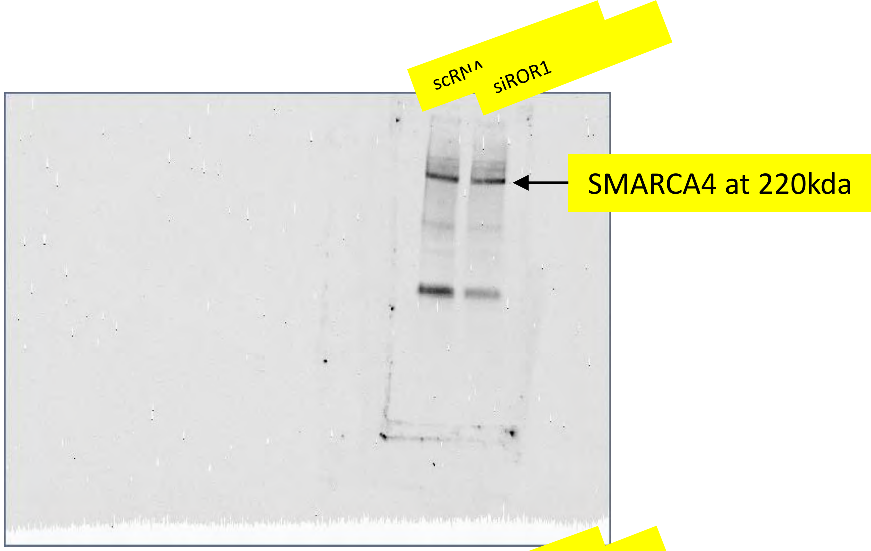

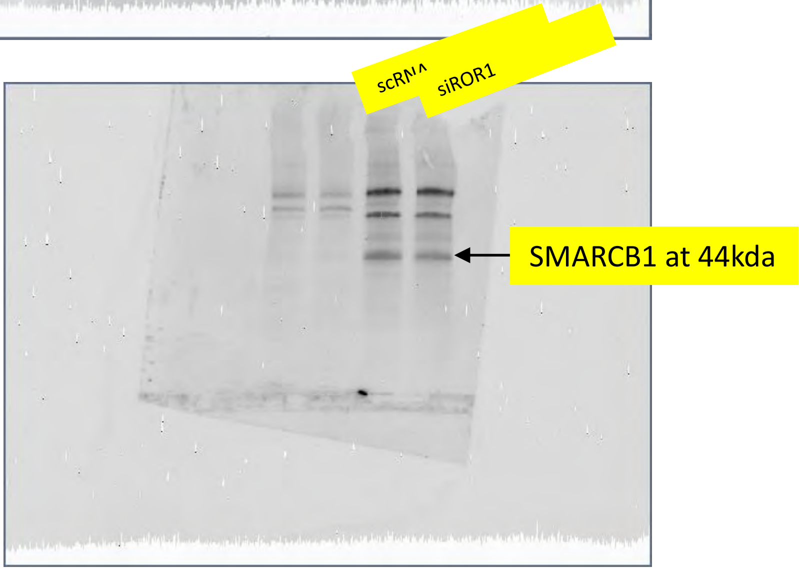

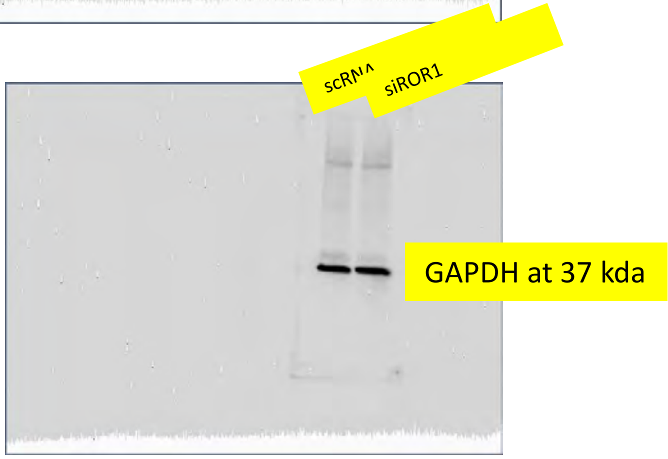

Supporting gel data figure S5B

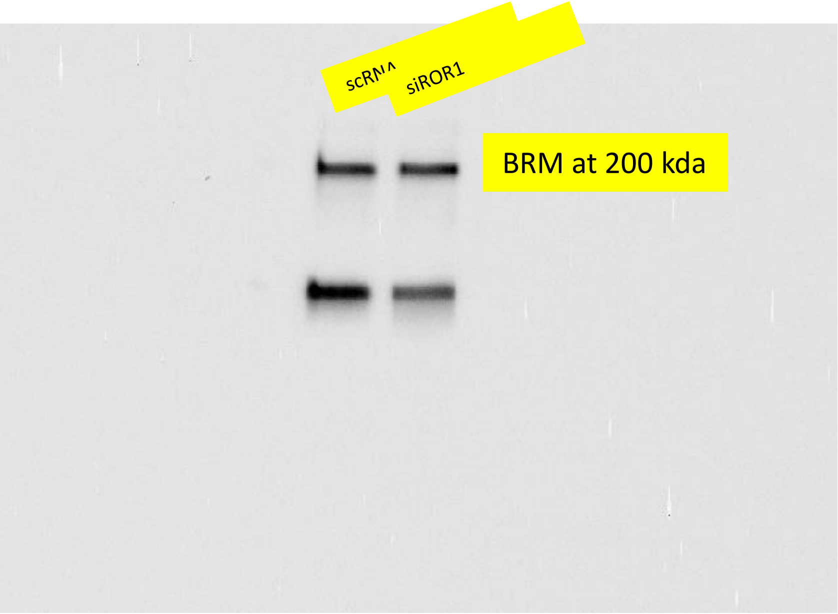

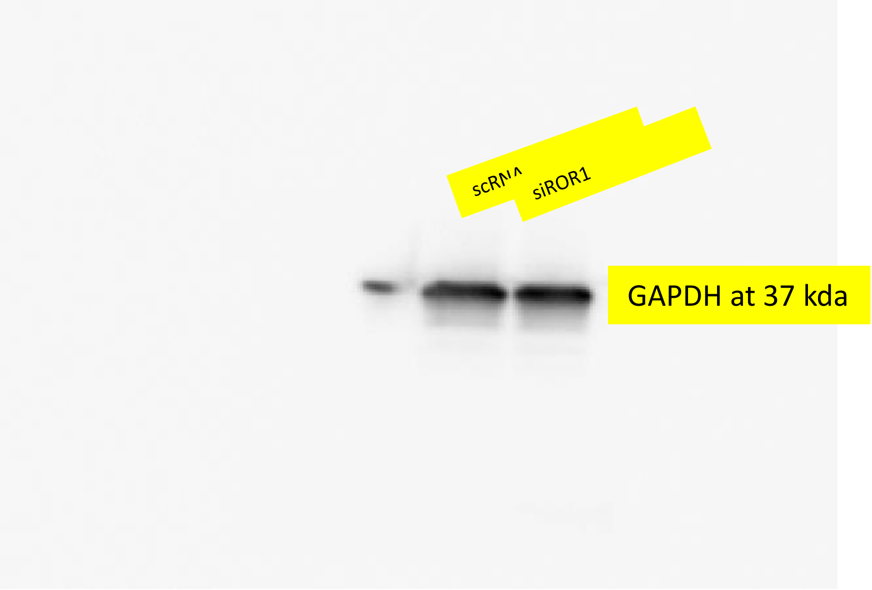

Figure S1. **ROR1 overexpression in ROR1-deficient MCF-7 decreases drug sensitivity A)** Immunoblot assessing efficacy of ROR1 knockdown via siRNA in SUM159PT/R **B)** Immunoblot assessing efficacy of ROR1 overexpression in ROR1-deficient MCF-7 luminal breast cancer line. Full length blots are presented in the supplementary figure S15 **C, D)** MTT investigating cell viability following Dox/Cis treatment in both cell lines after either ROR1 overexpression or Control vector transfection. All statistical analyses via student's t test. N = 3. * = p <0.05 , ** = p <0.01

Figure S2. ROR1 inhibition potentiates DNA damage induced by chemo drugs in SUM159PT A) Immunofluorescence labelling γH2A.X in SUM159PT transfected with either siROR1 or control RNA then treated with Dox or vehicle control (DMSO). **B)** ImageJ quantification of individual γH2A.X foci in nuclei from images obtained in (A). **C)** Overall nuclear γH2A.X

expression (mean fluorescence intensity), calculated using ImageJ, of cells imaged in **(A)**. Statistical analyses were performed using Student's t test. * = p <0.05, ** = p<0.01.

Figure S3. ROR1 inhibition represses ABCB1 expression in SUM-159PT A, B) Immunoblot assessing ABCB1 protein expression after ROR1 knockdown via siRNA (A) or ROR1 inhibition with StrC (B) in SuM-159PT. Full length blots are presented in the supplementary figure S16 and S17.

Figure S4. ROR1 overexpression in ROR1-deficient MCF-7 increases ABCB1 transcription A, B) qPCR investigating ROR1 and ABCB1 mRNA levels in ROR1-deficient MCF-7 following ROR1 overexpression. All statistical analyses via student's t test. N = 3. *** = p <0.001 , **** = p
<0.0001

Figure S5. ROR1 inhibition does not affect expression of SWI/SNF complex members A, B) Immunoblots assessing expression of other SWI/SNF complex members after ROR1 siRNA knockdown. Full length blots are presented in the supplementary figure S18 and S19.

Figure S6. ROR1 inhibition represses drug efflux in SUM159PT. A, C) Fluorescence confocal microscopy monitoring doxorubicin (red) in SUM159PT nuclei (DAPI/blue) following treatment, after ROR1 knockdown (A) or inhibition with StrC (C). **B,D)** ImageJ quantification of Doxorubicin within nuclei (mean fluorescence intensity) from images obtained in (A) and (C). Statistical analyses via student's t test. N = 3. p < 0.05 considered statistically significant.

Figure S7. Supporting full length blot data for Fig 1D

Figure S8. Supporting full length blot data for Fig 2A

Figure S9. Supporting full length blot data for Fig 4A

Figure S10. Supporting full length blot data for Fig 5B

Figure S11. Supporting full length blot data for Fig 5B

Figure S12. Supporting full length blot data for Fig 6B

Figure S13. Supporting full length blot data for Fig 6B

Figure S14. Supporting full length blot data for Fig 6C

Figure S15. Supporting full length blot data for Fig S1A

Figure S16. Supporting full length blot data for Fig S3A

Figure S17. Supporting full length blot data for Fig S3B

Figure S18. Supporting full length blot data for Fig S5A

Figure S19. Supporting full length blot data for Fig S5B
